# Customized Treatment in Non-Small-Cell Lung Cancer Based on EGFR Mutations and BRCA1 mRNA Expression

**DOI:** 10.1371/journal.pone.0005133

**Published:** 2009-05-05

**Authors:** Rafael Rosell, Laia Perez-Roca, Jose Javier Sanchez, Manuel Cobo, Teresa Moran, Imane Chaib, Mariano Provencio, Manuel Domine, Maria Angeles Sala, Ulpiano Jimenez, Pilar Diz, Isidoro Barneto, Jose Antonio Macias, Ramon de las Peñas, Silvia Catot, Dolores Isla, Jose Miguel Sanchez, Rafael Ibeas, Guillermo Lopez-Vivanco, Juana Oramas, Pedro Mendez, Noemi Reguart, Remei Blanco, Miquel Taron

**Affiliations:** 1 Catalan Institute of Oncology, Badalona, Spain; 2 Pangaea Biotech, USP Institut Universitari Dexeus, Barcelona, Spain; 3 Autonomous University of Madrid, Madrid, Spain; 4 Hospital Clinico Carlos Haya, Malaga, Spain; 5 Hospital Puerta de Hierro, Madrid, Spain; 6 Fundación Jimenez Diaz, Madrid, Spain; 7 Hospital Basurto, Bilbao, Spain; 8 Hospital de la Princesa, Madrid, Spain; 9 Complejo Hospitalario de Leon, Leon, Spain; 10 Hospital Reina Sofia, Cordoba, Spain; 11 Hospital Morales Meseguer, Murcia, Spain; 12 Hospital Provincial de Castellon, Castellon, Spain; 13 Althaia, Manresa, Spain; 14 Hospital Clinico Lozano Blesa, Zaragoza, Spain; 15 Hospital de Alcorcon, Madrid, Spain; 16 Hospital Municipal de Badalona, Badalona, Spain; 17 Hospital de Cruces, Barakaldo, Spain; 18 Hospital Universitario de Canarias, Tenerife, Spain; 19 Hospital Clinic, Barcelona, Spain; 20 Hospital de Terrassa, Barcelona, Spain; Ordway Research Institute, United States of America

## Abstract

**Background:**

Median survival is 10 months and 2-year survival is 20% in metastatic non-small-cell lung cancer (NSCLC) treated with platinum-based chemotherapy. A small fraction of non-squamous cell lung cancers harbor EGFR mutations, with improved outcome to gefitinib and erlotinib. Experimental evidence suggests that BRCA1 overexpression enhances sensitivity to docetaxel and resistance to cisplatin. RAP80 and Abraxas are interacting proteins that form complexes with BRCA1 and could modulate the effect of BRCA1. In order to further examine the effect of EGFR mutations and BRCA1 mRNA levels on outcome in advanced NSCLC, we performed a prospective non-randomized phase II clinical trial, testing the hypothesis that customized therapy would confer improved outcome over non-customized therapy. In an exploratory analysis, we also examined the effect of RAP80 and Abraxas mRNA levels.

**Methodology/Principal Findings:**

We treated 123 metastatic non-squamous cell lung carcinoma patients using a customized approach. RNA and DNA were isolated from microdissected specimens from paraffin-embedded tumor tissue. Patients with EGFR mutations received erlotinib, and those without EGFR mutations received chemotherapy with or without cisplatin based on their BRCA1 mRNA levels: low, cisplatin plus gemcitabine; intermediate, cisplatin plus docetaxel; high, docetaxel alone. An exploratory analysis examined RAP80 and Abraxas expression. Median survival exceeded 28 months for 12 patients with EGFR mutations, and was 11 months for 38 patients with low BRCA1, 9 months for 40 patients with intermediate BRCA1, and 11 months for 33 patients with high BRCA1. Two-year survival was 73.3%, 41.2%, 15.6% and 0%, respectively. Median survival was influenced by RAP80 expression in the three BRCA1 groups. For example, for patients with both low BRCA1 and low RAP80, median survival exceeded 26 months. RAP80 was a significant factor for survival in patients treated according to BRCA1 levels (hazard ratio, 1.3 [95% CI, 1–1.7]; P = 0.05).

**Conclusions/Significance:**

Chemotherapy customized according to BRCA1 expression levels is associated with excellent median and 2-year survival for some subsets of NSCLC patients , and RAP80 could play a crucial modulating effect on this model of customized chemotherapy.

**Trial Registration:**

ClinicalTrials.gov NCT00883480

## Introduction

The median survival of patients with advanced or metastatic non-small-cell lung cancer (NSCLC) is only 10–11 months with either standard cisplatin-based chemotherapy [1], [2] or customized cisplatin-based chemotherapy based on excision repair cross-complementing 1 (ERCC1) mRNA expression,[3] and the two-year survival rate is only 14–21%.[1], [2], [3]

The two proto-oncogenes currently known to be more commonly mutated in lung adenocarcinoma are K-RAS and EGFR[4]. Lung cancers caused by activating mutations in the epidermal growth factor receptor (EGFR) – mainly either deletion at exon 19 or L858R mutation at exon 21 – respond to small molecule tyrosine kinase inhibitors (gefitinib and erlotinib),[5], [6], [7] with a recently reported median survival to gefitinib of 17.5 months.[8] Response rate was 90% in our retrospective trial examining EGFR mutations in patients treated with gefitinib,[9] and pooled data of prospective trials of gefitinib in patients with EGFR mutations showed a response rate of 80%.[10] However, no EGFR mutations were found in 454 patients with squamous cell carcinoma of the lung.[11]

A growing body of evidence indicates that the breast cancer susceptibility gene 1 (BRCA1) confers sensitivity to apoptosis induced by antimicrotubule drugs (paclitaxel and vincristine) but induces resistance to DNA-damaging agents (cisplatin and etoposide) and radiotherapy.[12], [13], [14], [15] These pre-clinical findings are supported by a variety of experimental models in breast and ovarian cancer cells: inducible expression of BRCA1 enhanced paclitaxel sensitivity;[16] a short interfering RNA-mediated inactivation of endogenous BRCA1 led to paclitaxel and docetaxel resistance;[17], [18], [19] and reconstitution of BRCA1-deficient cells with wild-type BRCA1 enhanced sensitivity to paclitaxel and vinorelbine.[17] This differential modulating effect of BRCA1 mRNA expression was also observed in tumor cells isolated from malignant effusions of NSCLC and gastric cancer patients, where BRCA1 mRNA levels correlated negatively with cisplatin sensitivity and positively with docetaxel sensitivity.[20] Two retrospective studies – in NSCLC [21] and ovarian cancer[19] patients – found that low or intermediate BRCA1 mRNA levels correlated with a significantly longer survival following platinum-based chemotherapy,[19], [21] while survival in patients with higher BRCA1 expression increased following taxane-based chemotherapy.[19]

BRCA1 is recruited to the sites of DNA breaks, playing a central role in DNA repair and in cell-cycle checkpoint control. Binding of the mediator of DNA damage checkpoint 1 (MDC1) protein to the phosphorylated tail of histone H2AX facilitates the formation of BRCA1 nuclear foci at double-strand breaks.[22] The receptor-associated protein 80 (RAP80) acts upstream of BRCA1 and is required for the accumulation of BRCA1 to sites of DNA breaks.[23], [24], [25] Abraxas recruits RAP80 to form a complex with BRCA1. Both Abraxas and RAP80 are required for DNA damage repair, and cells depleted of Abraxas or RAP80 exhibit hypersensitivity to irradiation.[23]

In order to examine whether customizing treatment could improve outcome in advanced NSCLC patients, we have performed a prospective non-randomized phase II trial of customized treatment based on EGFR mutation status and BRCA1 mRNA expression levels. We opted to limit enrollment to non-squamous cell carcinoma in order to maximize the opportunity to administer erlotinib in patients with EGFR mutations. Patients with either the exon 19 deletion or the L858R mutation received erlotinib, while those with wild-type EGFR received chemotherapy based on BRCA1 levels: those with low levels received cisplatin plus gemcitabine; those with intermediate levels received cisplatin plus docetaxel; and those with high levels received docetaxel alone. In an exploratory analysis, we also examined the effect of RAP80 and Abraxas mRNA levels in these patients.

## Results

### Patients

**Figure 1:**
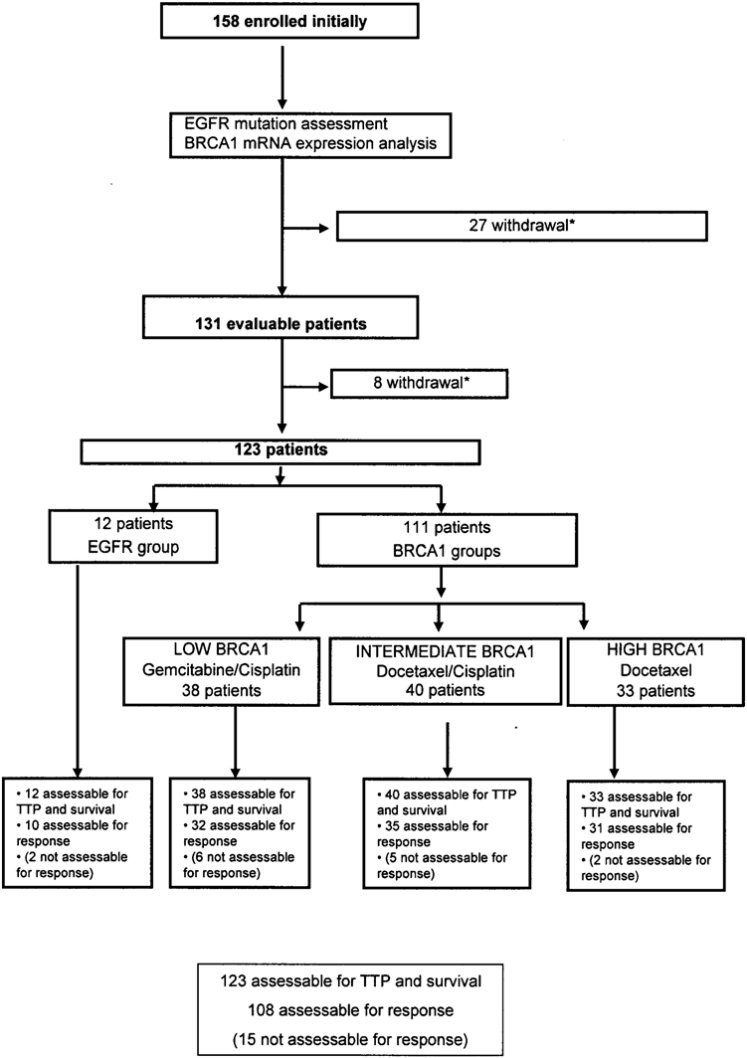
Consolidated Standards of Reporting Trials diagram showing flow of patients through study. Between March 2005 and July 2007, a total of 123 patients from 25 centers were enrolled in the study. Reasons for patient withdrawal: 3 patients had no tumor cells in the biopsy; 5 patients had less than 50 tumor cells in the biopsy, making it impossible to assure correct results; 19 patients were wild-type EGFR but with insufficient tumor sample after EGFR assessment for BRCA1 expression analysis; 2 patients refused to participate; and 6 patients were withdrawn by their physician due to clinical factors unrelated to the study. The two patients in the EGFR group who were not evaluable for response died within a month of entering the study; the 13 patients in the BRCA1 who were not evaluable for response received >3 cycles of treatment.

Between March 2005 and July 2007, a total of 123 patients from 25 centers were enrolled in the study (Figure 1). Thirty-five patients were excluded: 3 patients had no tumor cells in the biopsy; 5 patients had less than 50 tumor cells in the biopsy, making it impossible to assure correct results; 19 patients were wild-type EGFR but with insufficient tumor sample after EGFR assessment for BRCA1 expression analysis; 2 patients refused to participate; and 6 patients were withdrawn by their physician due to clinical factors unrelated to the study. For all 123 patients, RNA isolation and PCR amplification were successful. On average, results of genetic analyses were available in 8 days (range, 6–11 days). The median number of cycles of chemotherapy administered in the BRCA1 groups was 5 (range, 1.8). Median follow-up was 8 months (range, 1–28 months). Twelve patients had EGFR mutations and were assigned to receive erlotinib (EGFR group). Of the 111 patients with wild-type EGFR, 38 were in the lowest tercile of BRCA1 expression and were assigned to receive cisplatin plus gemcitabine (low BRCA1 group), 40 were in the intermediate tercile and were assigned to receive cisplatin plus docetaxel (intermediate BRCA1 group), and 33 were in the highest tercile and were assigned to receive docetaxel alone (high BRCA1 group) (Figure 1).

The clinical characteristics of the four groups are shown in Table 1 and Table S1. Median age for all patients was 60 years. Proportionally more females than males were in the lowest tercile of BRCA1 expression. EGFR mutations were more frequently observed in never-smokers (P = 0.03) and females (P = 0.0001). Fifty-five percent of patients had a performance status of 1, and 83% had stage IV disease. Seventeen percent of patients had brain metastases. Patients with EGFR mutations had a median of two metastatic sites, compared to one site in patients with wild-type EGFR (Table S2).

**Table 1:** Patient characteristics

		All patients	EGFR Group	BRCA1 Groups		
				Low	Intermediate	High
		N = 123	N = 12	N = 38	N = 40	N = 33
		N (%)	N (%)	N (%)	N (%)	N (%)
**Age**	Median(range)	60 (36–78)	60 (42–70)	60 (36–77)	58 (43–78)	60 (42–75)
**Gender**	Female	38 (30.9)	9 (75)	15 (39.5)	11 (27.5)	3 (9.1)
	Male	85 (69.1)	3 (25)	23 (60.5)	29 (72.5)	30 (90.9)
**Smoker**	Current	40 (32.5)	1 (8.3)	7 (18.4)	18 (45)	14 (42.4)
	Never	26 (21.1)	7 (58.3)	8 (21.1)	8 (20)	3 (9.1)
	Former	57 (46.4)	4 (33.4)	23 (60.5)	14 (35)	16 (48.5)
**Race**	Caucasian	122 (99.2)	12 (100)	38 (100)	39 (97.5)	33 (100)
	Other	1 (0.8)	0 (0)	0 (0)	1 (2.5)	0 (0)
**ECOG PS**	0	44 (35.8)	6 (50)	15 (41.7)	12 (30.7)	11 (33.3)
	1	68 (55.3)	5 (41.7)	20 (55.5)	23 (58.9)	20 (60.6)
	2	8 (6.5)	1 (8.3)	1 (2.8)	4 (10.4)	2 (6.1)
	NR	3 (2.4)	0	2	1	0
**Histology**	Adeno	83 (67.5)	8 (66.7)	27 (71.1)	27 (67.5)	21 (63.)
	BAC	10 (8.1)	3 (25)	5 (13.2)	2 (5)	0 (0)
	LCC	14 (11.4)	1 (8.3)	2 (5.3)	5 (12.5)	6 (18.2)
	NOS	16 (13)	0	4 (10.5)	6 (15)	6 (18.2)
**Stage**	III	21 (17.1)	3 (25)	10 (26.3)	5 (12.5)	3 (9.1)
	IV	102 (82.9)	9 (75)	28 (73.7)	35 (87.5)	30 (90.9)

ECOG, Eastern Cooperative Oncology Group; PS, performance status; NR, not recorded; adeno, adenocarcinoma; BAC, bronchioloalveolar carcinoma; LCC, large cell carcinoma; NOS, non-specified;

The overall response rate was 90% for the EGFR group, 25% for the low BRCA1 group, 45.7% for the intermediate BRCA1 group, and 41.9% for the high BRCA1 group (Table 2). In the intent-to-treat analysis, the response rate was 75% for the EGFR group, 21.1% for the low BRCA1 group, 40% for the intermediate BRCA1 group, and 39.4% for the high BRCA1 group (Table 2).

**Table 2:** Outcomes according to treatment groups

		All Patients		EGFR Group		BRCA1 Groups					
						Low		Intermediate		High	
		(n = 123)		(n = 12)		(n = 38)		(n = 40)		(n = 33)	
		%	95% CI	%	95% CI	%	95% CI	%	95% CI	%	95% CI
**Outcome**	CR	3.3		16.7		0		2.5		3	
	PR	34.1		58.3		21.1		37.5		36.4	
	SD	30.1		8.3		47.4		17.5		33.3	
	PD	20.3		0		15.8		30		21.2	
	ND	12.2		16.7		15.8		12.5		6.1	
**ORR**		43.6		90		25		45.7		41.9	
**Intent to treat**		37.4		75		21.1		40		39.4	
											
**Survival**	MS, mo	12 mo	8.5–15.5	NR (>28 mo)	-	11 mo	1–20.9	9 mo	5.4–12.6	11 mo	8.2–13.8
	1-yr	49.2	39.5–58.8	91.7	57.2–100	47.8	30.9–64.6	41.1	23.6–58.4	42.4	23.5–61.1
	2-yr	31.5	21.1–41.9	73.3	17.6–100	41.2	24.3–58	15.6	0–32.2	0	
	28 mo	24.5	12.7–36.3	73.3	17.6–100	35.3	17.4–53.1	0	-	0	
**TTP**		6 mo	4.2–7.7	13 mo	7.7–18.3	5 mo	2.7–7.3	5 mo	2.7–7.3	8 mo	5.1–10.9

ORR, overall response rate; TTP, time to progression; CR, complete response; PR, partial response; SD, stable disease; PD, progressive disease; ND, not determined; MS, median survival

**Figure 2:**
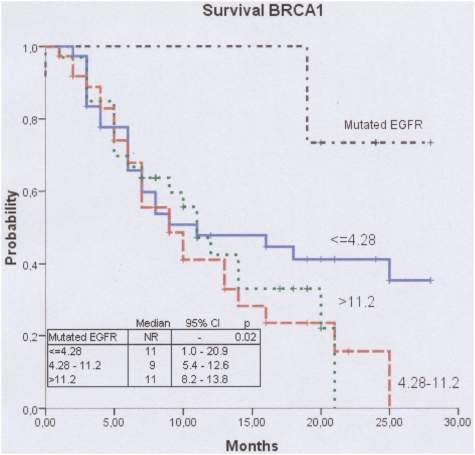
Median survival according to treatment group. Median survival was not reached for 12 patients in the EGFR group, 11 months for 38 patients in the low BRCA1 group, 9 months for 40 patients in the intermediate BRCA1 group, and 11 months for 33 patients in the high BRCA1 group (P = 0.01) (see Table 2).

Median survival was not reached but exceeded 28 months for the EGFR group, compared to 10 months (95% CI, 8.5 to 15–5) for patients in all three BRCA1 groups. Two-year survival for patients in the EGFR group was 73.3% and for all patients in the BRCA1 groups it was 26.7%. For patients in the low BRCA1 group, median survival was 11 months (95% CI, 1.1 to 20.9) and 2-year survival was 41.2%. For those in the intermediate BRCA1 group, median survival was 9 months (95% CI, 5.4 to 12.6) and 2-year survival was 15.6%. For patients in the high BRCA1 group, median survival was 11 months (95% CI, 8.2 to 13.8) and 2-year survival was 0% (Table 2, Figure 2).

Median time to progression was 13 months (95% CI, 7.7 to 18.3) in the EGFR group, compared to 6 months (95% CI, 4.7 to 7.2) for patients in all three BRCA1 groups. For patients in the low and intermediate BRCA1 groups, time to progression was 5 months (95% CI, 2.7 to 7.3). For patients in the high BRCA1 group, time to progression was 8 months (95% CI, 5.1 to 10.9) (Table 2, Figure S1).

### RAP80 and Abraxas mRNA transcripts

Based on the results of experimental models[23], [24], [25], an exploratory analysis of the relation between BRCA1, RAP 80 and Abraxas mRNA expression was performed in 86 of 111 patients without EGFR mutations for whom sufficient tumor tissue was available. Patient characteristics for these 86 patients were similar to those of all 111 patients; significantly more females than males had low BRCA1 expression (P = 0.009). Response was significantly higher in patients with intermediate and high BRCA1 levels (P = 0.004). (Table S3). A close correlation was found between BRCA1 and RAP 80 levels (ρ = 0.27; P = 0.02) and between RAP 80 and Abraxas levels (ρ = 0.41; P<0.001) but not between BRCA1 and Abraxas levels (ρ = 0.10; P = 0.39).

**Figure 3:**
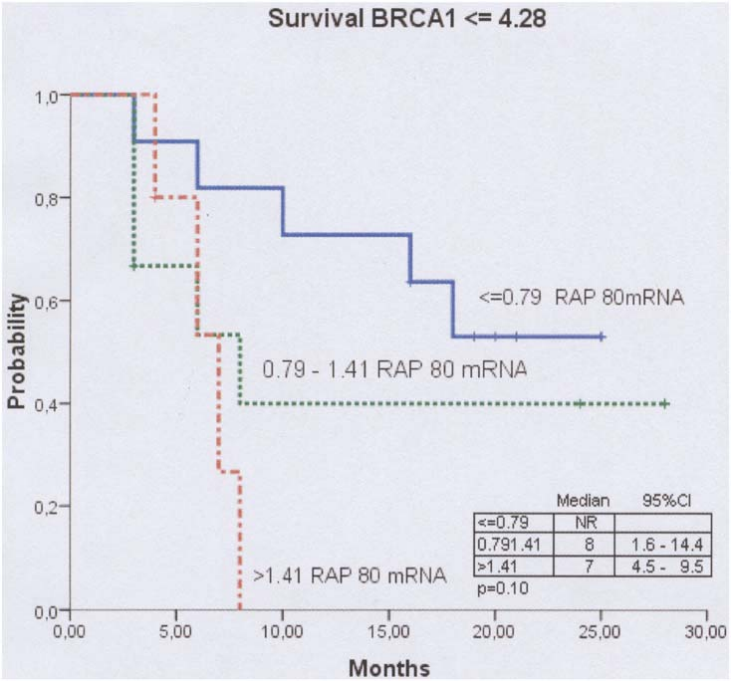
Median survival for patients with low BRCA1 levels, treated with cisplatin plus gemcitabine, according to RAP 80 mRNA expression. Median survival was not reached for 11 patients with low RAP 80 levels, 8 months for 9 patients with intermediate RAP 80 levels, and 7 months for 5 patients with high RAP 80 levels (P = 0.006).

Median survival was influenced by RAP 80 levels. In patients with low BRCA1 levels, median survival was not reached in patients with low RAP 80 levels, while it was 8 months for patients with intermediate RAP 80 and 7 months for those with high RAP 80 (Table 3, Figure 3). In patients with intermediate BRCA1 levels, median survival was 5 months in patients with low RAP 80 levels, while it was 13 months for patients with intermediate RAP 80 levels and 16 months for those with high RAP 80 levels. In patients with high BRCA1 levels, median survival was 6 months in patients with low RAP 80 levels, 12 months in patients with intermediate RAP80 levels, and 11 months for those with high RAP 80 levels (Table 3).

**Table 3:** Median survival according to levels of BRCA1 and RAP80

		RAP 80 LEVELS						
		≤0.79		0.79–1.41		>1.41		
BRCA1 Levels		N	months (95% CI)	N	months (95% CI)	N	months (95% CI)	P
	Low	11	NR (-)	9	8 (1.6–14.4)	5	7 (4.5–9.5)	0.10
	Intermediate	11	5 (3.4–6.6)	7	13 (10–15.9)	16	16 (5.5–26.5)	0.15
	High	5	6 (1.8–10.1)	9	12 (9.3–14.6)	12	11 (8.2–13.8)	0.17

CI, confidence interval; NR, not reached

In patients with low BRCA1 levels, time to progression was 14 months in patients with low RAP 80 levels, while it was 4 months for patients with intermediate RAP80 levels and 6 months for those with high RAP 80 levels (Table 4, Figure S2). In patients with intermediate BRCA1 levels, time to progression was 4 months in patients with low RAP 80 levels, while it was 9 months for patients with intermediate RAP 80 levels and 6 months for those with high RAP 80 levels. In patients with high BRCA1 levels, time to progression was 2 months in patients with low RAP 80 levels, 10 months in patients with intermediate RAP 80 levels, and 4 months for those with high RAP 80 levels (Table 4).

**Table 4:** Time to progression according to levels of BRCA1 and RAP 80

		RAP 80 LEVELS						
		≤0.79		0.79–1.41		>1.41		
BRCA1 Levels		N	months (95% CI)	N	months (95% CI)	N	months (95% CI)	P*
	Low	11	14 (5–22.9)	9	4 (2.8–5.1)	5	6 (-)	0.08
	Intermediate	11	4 (3.1–4.9)	7	9 (2.5–15.5)	9	6 (3.1–8.9)	0.42
	High	5	2 (0–4.1)	9	10 (7.3–12.6)	12	4 (1.7–6.3)	0.006

CI, confidence interval

* All p-values were corrected using the Bonferroni method.

Similar results were obtained when median survival and time to progression were compared according to Abraxas mRNA expression levels (Tables S4 and S5). An exploratory multivariate analysis in the 86 patients, with the use of a Cox proportional-hazards model, identified ECOG performance status and RAP 80 as significant variables for survival (hazard ratios: performance status 1, 2.72; P = 0.005; RAP 80, 1.3; P = 0.05) (Table S6). Survival was not influenced by other clinical characteristics, types of metastases, second-line chemotherapy (36 patients), or Abraxas levels. The Cox model for time to progression also showed that only performance status and RAP 80 were significant variables (Table S7).

## Discussion

Mutations in the EGFR tyrosine kinase domain induce lung adenocarcinoma in mice[26] and a favorable response to first- and second-line gefitinib and erlotinib in advanced NSCLC.[7], [8] In the present study, median survival exceeded 28 months in 12 patients with EGFR mutations treated with erlotinib, with a median time to progression of 13 months and a two-year survival of 73.3%; these results are similar to the findings of a meta-analysis of prospective trials with gefitinib in patients with EGFR mutations.[10] Median survival was 11 months in patients with the lowest BRCA1 expression, treated with cisplatin plus gemcitabine, and two-year survival was 41.2%, which compares favorably with the median and two-year survival attained with gemcitabine plus cisplatin or pemetrexed plus cisplatin (10.3 months and 22%) in a recent randomized trial.[2] In patients with the highest BRCA1 expression, treated with docetaxel alone, median survival was 11 months, identical to that obtained in a large phase III trial in patients treated with docetaxel plus cisplatin.[1] However, in our study, no patient was alive at two years, while in the phase III trial, two-year survival was 21%.[1] Intriguingly, 11 patients with the lowest expression of both BRCA1 and RAP 80 had an outcome similar to that attained by patients with EGFR mutations treated with erlotinib: median survival was not reached and time to progression was 14 months (Table 3, Figure 3).Chemotherapy response is solidly based on the fact that DNA repair genes require a series of molecular recognition steps that enable DNA damage response proteins to localize at and near DNA lesions. Binding of the mediator of DNA damage checkpoint 1 (MDC1) protein to the phosphorylated tail of histone H2AX (γH2AX) facilitates the formation of BRCA1 nuclear foci at double-strand breaks induced by irradiation or chemotherapy. By dimerizing with BRCA1-associated RING domain (BARD1) protein through the RING domain, BRCA1 forms an E3 ubiquitin ligase. Recently, it has been shown that RAP 80 targets the BRCA1-BARD1 E3 ligase to MDC1-γH2AX-dependent lysine 63-linked ubiquitin proteins at double-strand breaks (reviewed in Wang & Elledge[27]). Three studies showed that the abrogation of RAP 80 reduced the formation of BRCA1-induced foci to 28%,[23] 2%[24] and 0%.[25] Moreover, Abraxas and RAP 80 foci formation is BRCA1-independent.[23] We therefore hypothesized that if RAP 80 was elevated, it could cause resistance to cisplatin-based chemotherapy even in the presence of low BRCA1 levels. The exploratory assessment of RAP 80 in the present study confirms its modulating effect on the BRCA1 customized model. For example, median survival in patients with the lowest BRCA1 expression decreased as RAP 80 expression increased: 8 months with intermediate RAP 80 levels and 7 months with high RAP 80 levels (Table 3). Overexpression of BRCA1 confers sensitivity to docetaxel and paclitaxel;[12], [17], [19], [20] patients with the highest levels of BRCA1, treated with docetaxel, had a median survival of 11–12 months when RAP80 expression was also high but only 6 months when RAP 80 expression was low (Table 3). Patients with intermediate BRCA1 levels, treated with cisplatin plus docetaxel, had an overall median survival of 9 months, which increased to 13–16 months when RAP 80 levels were intermediate or high (Table 3). These results can be explained by pre-clinical findings that RAP 80 is able to translocate to irradiation-induced foci in HCC1937 cells which express a truncated BRCA1 that is unable to migrate to nuclear foci.[28] This indicates that RAP 80 could replace the BRCA1 DNA repair function in cells lacking BRCA1. Thus, although different platinum doublets show the same[29] – or slightly different[2] – survival overall, differences could be found when customizing chemotherapy based on a model of BRCA1 and RAP 80.

In the present study, no correlation was found between BRCA1 and Abraxas mRNA expression. However, there was an indication that expression levels of Abraxas modulate the effect of BRCA1. For example, patients with the lowest BRCA1 expression, treated with cisplatin plus gemcitabine, attained a median survival of 18 months and time to progression of 11 months when Abraxas levels were low (Table S4).

In addition to the potential predictive role of BRCA1, BRCA1 overexpression confers aggressive behavior in transgenic models of small cell and squamous cell lung carcinomas, as well as in a subset of lung adenocarcinomas harboring the intrinsic T/t-antigen cancer signature.[30] Poor prognosis has also been associated with BRCA1 overexpression in early NSCLC.[31] In the present study, two-year survival was 41% in patients with the lowest levels of BRCA1, 16% in those with intermediate levels and 0% in those with the highest levels.

The mechanisms of BRCA1 overexpression or downregulation in NSCLC remain to be clarified. However, it has recently been shown that DNA breaks swiftly activate heterochromatin protein 1-β (HP1-β), which promotes histone H2AX phosphorylation, initiating the BRCA1 signaling assembly for DNA repair.[32] Intriguingly, casein kinase 2 promotes the mobilization of HP1-β and is associated with poor prognosis in NSCLC.[33] While BRCA1 methylation is observed in ductal breast cancer, it is only found in 4% of NSCLCs.[34] Low BRCA1 expression in tumors may be due to the loss of histone methyltransferases, which leads to decreased chromatin H3 methylation in lysine 9, with the consequent downregulation of HP1-β.[35]

In this phase II, non-randomized study, the genetic factor and treatment difference are entirely co-existing, and caution should be exercised when interpreting the results. However, the exploratory analysis indicates that there is some evidence for tailoring chemotherapy based on BRCA1 and RAP 80 levels. Moreover, in a recent study of 96 stage IV NSCLC patients treated with docetaxel plus gemcitabine, we observed that as BRCA1 mRNA levels increased, the probability of response increased and the risk of progression decreased. For patients with the highest BRCA1 levels, the response rate was 58.6%, compared to 13.8% for those with intermediate levels and 27.6% for those with the lowest levels.[36] Based on these findings and those of the present study, the Spanish Lung Cancer Group is now modifying the protocol for a planned international phase III trial in advanced NSCLC to include customization based on RAP 80 as well as BRCA1 mRNA expression. Patients in the control arm will receive cisplatin plus docetaxel and those in the experimental arm will receive chemotherapy based on RAP 80 and BRCA1 mRNA levels: low RAP 80 levels (regardless of BRCA1 levels), cisplatin plus gemcitabine; intermediate or high RAP 80 and low or intermediate BRCA1, cisplatin plus docetaxel; intermediate or high RAP 80 and high BRCA1, docetaxel alone.

## Materials and Methods

The protocol for this trial is available as supporting information; see Protocol S1 and Protocol S2.

### Ethics statement

The protocol was approved by each center's institutional ethics review board, and all patients provided written informed consent before enrollment.

### Patients

We recruited patients to this phase II prospective multicenter trial based on screening of EGFR mutations followed by BRCA1 mRNA expression analysis in paraffin-embedded tumor tissue. Clinical eligibility included stage IIIB with pleural effusion or stage IV NSCLC, measurable disease by Response Evaluation Criteria in Solid Tumors (RECIST), performance status 0–2 by Eastern Cooperative Oncology Group (ECOG) criteria, adequate hematologic, renal and hepatic function. Brain metastases were allowed. Patients with squamous cell tumors, prior systemic therapy for advanced NSCLC, or other clinically significant cancers within five years were not eligible.

Patients with EGFR mutations – either the exon 19 deletion or the L858R mutation – received 150 mg of daily oral erlotinib continuously until progression or intolerable adverse effects. Each cycle was 28 days. Patients with wild-type EGFR received customized chemotherapy based on BRCA1 mRNA levels. Patients in the lowest tercile of BRCA1 expression received cisplatin 75 mg/m^2^ on day 1 plus gemcitabine 1250 mg/m^2^ on days 1 and 8. Patients in the intermediate tercile received cisplatin 75 mg/m^2^ on day 1 plus docetaxel 75 mg/m^2^ on day 1. Patients in the highest tercile received docetaxel 75 mg/m^2^ on day 1. All chemotherapy was repeated every three weeks for a maximum of six cycles unless there was earlier evidence of disease progression or intolerable adverse effects.

Baseline assessment included a medical history, physical examination and tumor measurements of palpable lesions as well as lesions assessed by computed tomography scans. The baseline assessment method was repeated every other cycle, and then every six weeks until disease progression.

### Molecular analyses

#### Tumor tissue collection and laser capture microdissection

BRCA1, RAP80 and Abraxas gene expression and EGFR mutations were analyzed in RNA and DNA isolated from paraffin-embedded tumor tissues. For each tumor sample a haematoxylin/eosin stained slice was analyzed by our pathologist to select the tumor area. Two 5-micron slices were mounted on special slides (Pem-Membrane slides, Palm, Oberlensheim, Germany) for laser capture microdissection (CAPmover Microdissector, Carl Zeiss Microimaging, Barcelona, Spain) to ensure a minimum of 90% of tumor cells. One slide was used for RNA isolation and the second was used for DNA isolation.

#### Gene expression analysis

Gene expression analysis was performed in RNA isolated from the tumor tissue specimens. cDNA was synthesized using M-MLV retrotranscriptase enzyme. Template cDNA was added to Taqman Universal Master Mix (AB; Applied Biosystems, Foster City, CA, USA) with specific primers and probe for each gene (Table S8). The primer and probe sets were designed using Primer Express 2.0 Software (AB) and the RefSeq sequences (http://www.ncbi.nlm.nih.gov/entrez/query.fcgi?db=gene). Quantification of gene expression was performed using the ABI Prism 7900HT Sequence Detection System (AB).

#### EGFR mutation analysis

For isolation of DNA from microdissected tissue, the material was incubated with proteinase K and DNA was extracted with phenol-chloroform and ethanol precipitation. Primers for PCR amplification in nested reactions for exons 19 and 21 of EGFR are shown in the supporting information. Mutations were analyzed using two methods: DNA sequencing and length analysis of fluorescently labeled PCR products for EGFR deletions in exon 19, and sequencing and 5′ nuclease activity assay (TaqMan) for EGFR mutation in exon 21 (L858R).

(For further details on the molecular analyses, see Text S1).

### Statistical analysis

Patients were divided into groups based on terciles of BRCA1 expression since this division is less susceptible to bias in multiple comparisons. Cut-off points for the BRCA1 terciles were obtained from the Spanish Lung Cancer Group data base, which includes clinical and genetic characteristics of more than 600 Spanish lung cancer patients. Responses were recorded according to the RECIST criteria. Median time to progression and overall survival were calculated from the start of treatment to the first documented disease progression or death, respectively.

In order to compare quantitative variables among patients in each of the treatment groups, to explore associations between variables within each group, and to study the potential association between baseline characteristics and response, we used parametric tests (student's t-test or ANOVA) or their equivalent non-parametric tests (U Mann-Whitney, Kruskall Wallis) when normality did not hold. The normality of continuous variables was checked with the Kolmogorov-Smirnov test. In order to compare categorical variables and response percentages with their 95% CIs among treatment groups, we used either the two-sided Fisher's exact test or the Chi-square test.

The association of risk factors with time-to-event endpoints was analyzed with the two-sided logrank test, and the Kaplan-Meier method was used to plot the corresponding time-to-progression and survival curves. A univariate Cox regression analysis, with hazard ratios and their 95% CIs was used to assess the association between each potential prognostic factor and survival and time to progression. These factors were then included in a multivariate Cox proportional hazards regression model to evaluate the independent significance of each variable on survival and time to progression. The likelihood ratio test was used to assess the goodness of fit, and the Wald's test was used to assess the coefficient significance. For potential multiple comparisons, the p-values were corrected with the Bonferroni correction.

Eighty-six of the 111 patients without EGFR mutations for whom sufficient tumor tissue was available were included in an exploratory sub-analysis of the relation between BRCA1, RAP80 and Abraxas expression. Spearman's rank test was used to evaluate the correlation between BRCA1, RAP80 and ABRAXAS mRNA expression.

All statistical calculations were performed with the SPSS software statistical package, version 15.0 (SPSS, Inc., Chicago, IL, USA) and S-PLUS 6.1. Two-sided p-values of less than 0.05 were considered significant.

## Supporting Information

Text S1 Supplemental text
(0.04 MB DOC)

Table S1 Clinicopathological characteristics, gene expression levels, EGFR mutation status, and outcomes for all patients
(0.07 MB XLS)

Table S2 Types of metastases
(0.05 MB DOC)

Table S3 Characteristics of 86 patients in whom RAP 80 and Abraxas were analyzed
(0.07 MB DOC)

Table S4 Median survival according to levels of BRCA1 and Abraxas
(0.03 MB DOC)

Table S5 Time to progression according to levels of BRCA1 and Abraxas
(0.03 MB DOC)

Table S6 Multivariable COX model for survival with BRCA1 and RAP 80 as continuous variables
(0.03 MB DOC)

Table S7 Multivariable COX model for time to progression with BRCA1 and RAP 80 as continuous variables
(0.03 MB DOC)

Table S8 Primers and probes used in gene expression analyses
(0.04 MB DOC)

Figure S1 Time to progression according to treatment group. Time to progression was 13 months in the EGFR group, 5 months in the low and intermediate BRCA1 groups, and 8 months in the high BRCA1 group (see Table 2).
(8.92 MB TIF)

Figure S2 Time to progression for patients in the low BRCA1 group according to RAP 80 expression levels. Time to progression was 14 months for patients with low RAP 80 levels, 4 months for those with intermediate RAP 80 levels, and 6 months for those with high RAP 80 levels (see Table 4).
(10.31 MB TIF)

Protocol S1 Trial Protocol
(1.12 MB PDF)

Protocol S2 English summary of protocol
(0.21 MB PDF)
