# Supplementary material for: Customized Treatment in Non-Small-Cell Lung Cancer Based on EGFR Mutations and BRCA1 mRNA Expression
Source: PLoS One. 2009 May 5;4(5):e5133. doi: 10.1371/journal.pone.0005133 (PMC2673583; doi:10.1371/journal.pone.0005133)
Supplement: Text S1 — Supplemental text (0.04 MB DOC) [file pone.0005133.s001.doc]

**Text S1**

**Further details of molecular analyses**

***Gene expression analyses***

Gene expression analyses were performed in RNA isolated from the tumour tissue specimens, according to a proprietary procedure (application number 06807415 in Europe, application number 12/090767 in US, application number PCT/EP2006/067591 in the national phase in Australia and application number 2626533 in Canada) of Pangaea Biotech SA. Briefly, after tissue microdissection samples were lysed in a tris-chloride, EDTA, sodium dodecyl sulphate (SDS) and proteinase K containing buffer. RNA was extracted with phenol-chloroform-isoamyl alcohol followed by precipitation with isopropanol in the presence of glycogen and sodium acetate. RNA was resuspended in water and treated with DNAse I (Ambion Inc; Austin TX, USA) to avoid DNA contamination. Template cDNA was added to Taqman Universal Master Mix (AB) in a 12.5-μl reaction with specific primers and probe for each gene (Table S1).

Relative gene expression quantification was calculated according to the comparative Ct method using β-actin as an endogenous control and commercial RNA controls (Stratagene, La Jolla, CA) as calibrators. Final results were determined as follows: 2^-(ΔCt sample-ΔCt calibrator)^, where ΔC_T_ values of the calibrator and sample are determined by subtracting the C_T_ value of the target gene from the value of the β-actin gene. In all experiments, only triplicates with a standard deviation of the Ct value <0.20 were accepted. In addition, for each sample analyzed, a retrotranscriptase minus control was run in the same plate to assure lack of genomic DNA contamination.

***EGFR Mutational Analysis***

Primers for PCR amplification in nested reactions for exons 19 and 21 of EGFR were as follows: exon 19 (first PCR, forward 5’-GCAATATCAGCCTTAGGTGCGGCTC-3’ and reverse 5’-CATAGAAAGTGAACATTTAGGATGTG-3’;

nested PCR, forward 5’-GTGCATCGCTGGTAACATCC-3’ and reverse

5’-TGTGGAGATGAGCAGGGTCT-3’); exon 21 (first PCR, forward 5’-CTAA

CGTTCGCCAGCCATAAGTCC-3’ and reverse 5’-GCTGCGAGCTCACCCAGAATGTCTGG-3’; nested PCR, forward 5’-GCTCAGAGCCTGGCATGAA-3’ and reverse 5’-CATCCTCCCCTGCATGTGT-3’).

The first PCR was performed in a 50-μL reaction adding 2μL of sample, 2 unit of Ecotaq Polimerase (Ecogen, Barcelona, Spain), 7.5 μL of PCR buffer x10, 250 μM dNTPs, 3.5 mM MgCl2 and 0.5 pmol of each primer.

***Sequencing***

Sequencing was done using forward and reverse nested primers with the ABI Prism

3130 Genetic Analyzer (AB). Electropherograms were analyzed for the presence of mutations using Seqscape v2.1.1 software in combination with Factura to mark heterozygous positions. The human NSCLC cell line (PC9) (Kyushu Cancer Center, Fukuoka, Japan) was included as positive control for exon 19 deletion; H1975 cell line (American Type Culture Collection) was included as positive control for exon 21 L858R.

***Length analysis of fluorescently labelled PCR products for EGFR deletions in exon 19***

The products of the first PCR for exon 19 were amplified with the following primers: forward 5’-ACTCTGGATCCCAGAAGGTGAG-3’ and reverse 5’-FAM-CCACACAGCAAAGCAGAAACTC-3’. Amplification was done in a 50-μL reaction adding 1 unit of Ecotaq Polymerase (Ecogen), 250 μM dNTPs, 1 mM MgCl2 and 0.5 pmol of each primer. One μl of a 1/50 to 1/200 dilution of each PCR product was mixed with 0.5 μL of size standard (AB) and denatured in 9 μL formamide at 90ºC for 5 minutes. Separation was done with a four-color laser-induced fluorescence capillary electrophoresis system (ABI Prism 3130 Genetic Analyzer, AB). The collected data were evaluated with the GeneScan Analysis Software (Applera, Norwalk, CT). DNA from the PC-9 cell line was used as a positive control for exon 19 deletions.

***TaqMan assay for EGFR mutation in exon 21 (L858R)***

The products of the first PCR for exon 21 were analyzed by TaqMan with the following primers and probes: exon 21 (forward primer, 5’-AACACCGCAGCATGTCAAGA-3’, reverse primer 5’-TTCTCTTCCGCACCCAGC-3’; probes 5’-FAM-CAGATTTTGGGCGGGCCAAAC-TAMRA-3’ and 5’-VIC-TCACAGATTTTGGGCTGGCCAAAC-TAMRA-3’) Amplification was performed in a 25-μL reaction adding 2 μL of first PCR product, 12.5 μL of Ampli Taq Gold PCR Master Mix (AB), 0.6 pmol of each primer and 0.2 pmol of probes. Samples were analyzed in an ABI Prism 7000 Sequence Detection System (AB). DNA from the H1975 cell line was used as a positive control for the exon 21 mutation.

**Supplementary Figure Legends**

**Figure S1.** Time to progression according to treatment group. Time to progression was 13 months in the EGFR group, 5 months in the low and intermediate BRCA1 groups, and 8 months in the high BRCA1 group (see Table 2).

**Figure S2.** Time to progression for patients in the low BRCA1 group according to RAP 80 expression levels. Time to progression was 14 months for patients with low RAP 80 levels, 4 months for those with intermediate RAP 80 levels, and 6 months for those with high RAP 80 levels (see Table 4).
