# Supplementary material for: Customized Treatment in Non-Small-Cell Lung Cancer Based on EGFR Mutations and BRCA1 mRNA Expression
Source: PLoS One. 2009 May 5;4(5):e5133. doi: 10.1371/journal.pone.0005133 (PMC2673583; doi:10.1371/journal.pone.0005133)
Supplement: Table S2 — Types of metastases (0.05 MB DOC) [file pone.0005133.s003.doc]

**Table S2**. Types of metastases

| Metastasis Site | All Patients | EGFR Group | BRCA1 Group | | |  |
| --- | --- | --- | --- | --- | --- | --- |
|  |  |  | Low | Intermediate | High N=33 | P |
|  | N=123 | N=12 | N=38 | N=40 |  |  |
|  | N (%) | N (%) | N (%) | N (%) | N (%) |  |
| Adrenal | 12 (9.8) | 0 (0) | 4 (10.5) | 6 (15) | 2 (6.1) | 0.38 |
| Pleura | 6 (4.9) | 2 (16.7) | 2 (5.3) | 2 (55) | 0 (0) | 0.15 |
| Brain | 21 (17.1) | 4 (33.3) | 2 (5.3) | 8 (20) | 7 (21.2) | 0.08 |
| Lung | 48 (39) | 8 (66.7) | 14 (36.8) | 12 (30) | 14 (42.4) | 0.14 |
| Lymph nodes | 13 (10.6) | 4 (33.3) | 1 (2.6) | 4 (10) | 4 (12.1) | 0.03 |
| Bone | 18 (14.6) | 3 (25) | 7 (18.4) | 7 (17.5) | 1 (3) | 0.15 |
| Skin | 5 (4.1) | 0 (0) | 1 (2.6) | 3 (7.5) | 1 (3) | 0.57 |
| Liver | 10 (8.1) | 1 (8.3) | 3 (7.9) | 4 (10) | 2 (6.1) | 0.94 |
| Other | 7 (5.7) | 0 (0) | 2 (5.3) | 3 (7.5) | 2 (6.1) | 0.80 |
| No. metastases | 1 (0-7) | 2 (0-3) | 1 (0-3) | 1 (0-7) | 1 (0-3) | 0.05 |
