# Supplementary material for: Customized Treatment in Non-Small-Cell Lung Cancer Based on EGFR Mutations and BRCA1 mRNA Expression
Source: PLoS One. 2009 May 5;4(5):e5133. doi: 10.1371/journal.pone.0005133 (PMC2673583; doi:10.1371/journal.pone.0005133)
Supplement: Table S3 — Characteristics of 86 patients in whom RAP 80 and Abraxas were analyzed (0.07 MB DOC) [file pone.0005133.s004.doc]

**Table S3.** Characteristics of 86 patients in whom RAP 80 and Abraxas were analyzed

|  |  | All patients | BRCA1 | | |  |
| --- | --- | --- | --- | --- | --- | --- |
|  |  | N=86 | Low | Intermediate | High | P* |
|  |  |  | N=28 | N=29 | N=29 |  |
|  |  | N (%) | N (%) | N (%) | N (%) |  |
| **Age** | Median (range) | 60 (40-78) | 60 (40-77) | 59 (43-78) | 61 (42-75) | 0.53 |
| **Gender** | Female | 18 (20.9) | 11 (39.3) | 5 (17.2) | 2 (6.9) | 0.009 |
|  | Male | 68 (79.1) | 17 (60.7) | 24 (82.8) | 27 (93.1) |  |
| **Smoker** | Current | 30 (34.9) | 6 (21.4) | 14 (48.3) | 10 (34.5) | 0.64 |
|  | Never | 10 (11.6) | 5 (17.9) | 2 (6.9) | 3 (10.3) |  |
|  | Former | 46 (53.4) | 18 (58) | 13 (44.7) | 16 (54.1) |  |
| **Race** | Caucasian | 85 (98.8) | 28 (100) | 28 (96.6) | 29 (100) | 0.37 |
|  | Other | 1 (1.2) | 0 (0) | 1 (3.4) | 0 (0) |  |
| **ECOG PS** | 0 | 30 (34.9) | 15 (48.4) | 10 (32.3) | 10 (32.3) | 0.76 |
|  | 1 | 50 (58.1) | 15 (48.4) | 18 (58.1) | 19 (61.3) |  |
|  | 2 | 6 (7) | 1 (3.6) | 3 (10.3) | 2 (6.9) |  |
| **Histology** | Adeno | 56 (65.1) | 21 (75) | 18 (62.1) | 17 (58.6) | 0.16 |
|  | BAC | 4 (4.7) | 3 (10.7) | 1 (3.4) | 0 (0) |  |
|  | LCC | 11 (12.8) | 2 (7.1) | 3 (10.3) | 6 (20.7) |  |
|  | NOS | 15 (17.4) | 2 (7.1) | 7 (24.1) | 6 (20.7) |  |
| **Stage** | III | 16 (18.6) | 6 (20.5) | 5 (17.2) | 5 (17.2) | 0.87 |
|  | IV | 70 (81.4) | 22 (78.6) | 24 (82.8) | 24 (82.8) |  |
| **No. of cycles ccccccccccy9ccycycycles** |  | 5 (1-8) | 4 (1-8) | 4 (1-6) | 4 (1-8) | 0.26 |
| **Response** | CR | 1 (1.2) | 0 (0) | 1 (3.4) | 0 (0) | 0.004 |
|  | PR | 32 (39.5) | 6 (24) | 14 (48.3) | 12 (44.4) |  |
|  | CR+PR | 33 (40.7) | 6 (24) | 15 (51.7) | 12 (44.4) |  |
|  | SD | 28 (34.6) | 16 (64) | 3 (10.3) | 9 (33.3) |  |
|  | PD | 20 (24.7) | 3 (12) | 11 (37.9) | 6 (22.2) |  |
|  | NE | 5 |  |  |  |  |

*All p-values were corrected using the Bonferroni method.

ECOG, Eastern Cooperative Oncology Group; PS, performance status; adeno, adenocarcinoma; BAC, bronchioloalveolar carcinoma; LCC, large cell carcinoma; NOS, non-specified; CR, complete response ; PR, partial response ; SD, stable disease ; PD, progressive disease ; NE, not evaluated
