# Supplementary material for: Customized Treatment in Non-Small-Cell Lung Cancer Based on EGFR Mutations and BRCA1 mRNA Expression
Source: PLoS One. 2009 May 5;4(5):e5133. doi: 10.1371/journal.pone.0005133 (PMC2673583; doi:10.1371/journal.pone.0005133)
Supplement: Table S4 — Median survival according to levels of BRCA1 and Abraxas (0.03 MB DOC) [file pone.0005133.s005.doc]

**Table S4**. Median survival according to levels of BRCA1 and Abraxas

|  |  | Abraxas Levels | | | | | | |
| --- | --- | --- | --- | --- | --- | --- | --- | --- |
|  |  | ≤0.88 | | 0.88-2.13 | | >2.13 | |  |
|  |  | N | months (95% CI) | N | months (95% CI) | N | months (95% CI) | P* |
| BRCA1 Levels | Low | 8 | 18 (0-43.3) | 10 | NR (-) | 6 | 4 (0.4-7.6) | 0.03 |
|  | Intermediate | 7 | 6 (3.4-8.6) | 6 | 21 (-) | 9 | 7 (4.1-9.9) | 0.09 |
|  | High | 8 | 10 (4.4-15.5) | 7 | 20 (3.2-36.8) | 9 | 14 (2.6-25.3) | 0.35 |

CI, confidence interval; NR, not reached

*All p-values were corrected using the Bonferroni method.
