# Supplementary material for: Customized Treatment in Non-Small-Cell Lung Cancer Based on EGFR Mutations and BRCA1 mRNA Expression
Source: PLoS One. 2009 May 5;4(5):e5133. doi: 10.1371/journal.pone.0005133 (PMC2673583; doi:10.1371/journal.pone.0005133)
Supplement: Table S5 — Time to progression according to levels of BRCA1 and Abraxas (0.03 MB DOC) [file pone.0005133.s006.doc]

**Table S5**. Time to progression according to levels of BRCA1 and Abraxas

|  |  | Abraxas Levels | | | | | | |
| --- | --- | --- | --- | --- | --- | --- | --- | --- |
|  |  | ≤0.88 | | 0.88-2.13 | | >2.13 | |  |
|  |  | N | months (95% CI) | N | months (95% CI) | N | months (95% CI) | P |
| BRCA1 Levels | Low | 8 | 11 (0-24.5) | 10 | 7 (0-19.4) | 6 | 3 (0.4-5.6) | 0.32 |
|  | Intermediate | 7 | 4 (3.4-4.6) | 6 | 7 (2.8-11.2) | 9 | 5 (0-13.8) | 0.35 |
|  | High | 8 | 3 (0-7.1) | 7 | 5 (2.4-7.6) | 9 | 8 (3.3-12.7) | 0.67 |

CI, confidence interval
