# Supplementary material for: Customized Treatment in Non-Small-Cell Lung Cancer Based on EGFR Mutations and BRCA1 mRNA Expression
Source: PLoS One. 2009 May 5;4(5):e5133. doi: 10.1371/journal.pone.0005133 (PMC2673583; doi:10.1371/journal.pone.0005133)
Supplement: Table S6 — Multivariable COX model for survival with BRCA1 and RAP 80 as continuous variables (0.03 MB DOC) [file pone.0005133.s007.doc]

**Table S6**. Multivariable COX model for survival with BRCA1 and RAP 80 as continuous variables

|  |  | Hazard Ratio | 95% CI | P* |
| --- | --- | --- | --- | --- |
| **ECOG PS** | 0 | 1 (ref.) |  |  |
|  | 1 | 2.72 | 1.35-5.48 | 0.005 |
|  | 2 | 8.7 | 2.85-26.68 | <0.0001 |
| **Bone metastasis** | No | 1 (ref.) |  |  |
|  | Yes | 2.1 | 0.78-5.66 | 0.14 |
| **BRCA1** |  | 1.02 | 0.99-1.06 | 0.16 |
| **RAP 80** |  | 1.3 | 1.-1.7 | 0.05 |
| **BRCA1*RAP 80** |  | 0.98 | 0.96-1.01 | 0.28 |

CI, confidence interval; ECOG, Eastern Cooperative Oncology Group; PS, performance status

*All p-values were corrected using the Bonferroni method.
