# Supplementary material for: Customized Treatment in Non-Small-Cell Lung Cancer Based on EGFR Mutations and BRCA1 mRNA Expression
Source: PLoS One. 2009 May 5;4(5):e5133. doi: 10.1371/journal.pone.0005133 (PMC2673583; doi:10.1371/journal.pone.0005133)
Supplement: Table S7 — Multivariable COX model for time to progression with BRCA1 and RAP 80 as continuous variables (0.03 MB DOC) [file pone.0005133.s008.doc]

**Table S7**. Multivariable COX model for time to progression with BRCA1 and RAP 80 as continuous variables

|  |  | Hazard Ratio | 95% CI | P |
| --- | --- | --- | --- | --- |
| **ECOG PS** | 0 | 1 (ref.) |  |  |
|  | 1 | 1.4 | 0.77-2.40 | 0.27 |
|  | 2 | 3.6 | 1.29-10.52 | 0.01 |
| **Bone metastasis** | No | 1 (ref.) |  |  |
|  | Yes | 1.75 | 0.71-4.34 | 0.22 |
| **BRCA1** |  | 1.01 | 0.98-1.05 | 0.27 |
| **RAP 80** |  | 1.41 | 1.07-1.84 | 0.01 |
| **BRCA1*RAP 80** |  | 0.98 | 0.96-1.01 | 0.24 |

CI, confidence interval; ECOG, Eastern Cooperative Oncology Group; PS, performance status
