# Supplementary material for: Customized Treatment in Non-Small-Cell Lung Cancer Based on EGFR Mutations and BRCA1 mRNA Expression
Source: PLoS One. 2009 May 5;4(5):e5133. doi: 10.1371/journal.pone.0005133 (PMC2673583; doi:10.1371/journal.pone.0005133)
Supplement: Table S8 — Primers and probes used in gene expression analyses (0.04 MB DOC) [file pone.0005133.s009.doc]

**Table S8.** Primers and probes used in gene expression analyses

| Gene | RefSeq | Primer Forward (FW)/ Primer Reverse (RV)/ Probe (P) |
| --- | --- | --- |
| β-actin | NM_001101 | FW 5’ TGA GCG CGG CTA CAG CTT 3’ |
|  |  | RV 5’ TCC TTA ATG TCA CGC ACG ATT T 3’ |
|  |  | P 6FAM 5’ ACC ACC ACG GCC GAG CGG 3’ TAMRA |
| BRCA1 | NM_007294 | FW 5’ GGC TAT CCT CTC AGA GTG ACA TTT TA 3’ |
|  |  | RV 5’ GCT TTA TCA GGT TAT GTT GCA TGG T 3’ |
|  |  | P 6FAM 5’ CCA CTC AGC AGA GGG 3’ MGB |
| RAP80 | NM_016290 | FW 5´ ACATCAAGTCTTCAGAAACAGGAGC 3´ |
|  |  | RV 5’ TGCAGCCTGCCTCTTCCAT 3´ |
|  |  | P 6FAM 5’ TCAGGGTGCCTTCACCA 3´ MGB |
| Abraxas | NM_139076 | FW 5’ TGGTTTTAGCCGAGCAGTACAA 3’ |
|  |  | RV 5’ CTCCTTTAAGGATCCATCTTCTTCAA 3’ |
|  |  | P 6FAM 5’ ACACAGCTCTAAATTT 3’ MGB |
