# Supplementary figures and images for: Customized Treatment in Non-Small-Cell Lung Cancer Based on EGFR Mutations and BRCA1 mRNA Expression
Source: PLoS One. 2009 May 5;4(5):e5133. doi: 10.1371/journal.pone.0005133 (PMC2673583; doi:10.1371/journal.pone.0005133)

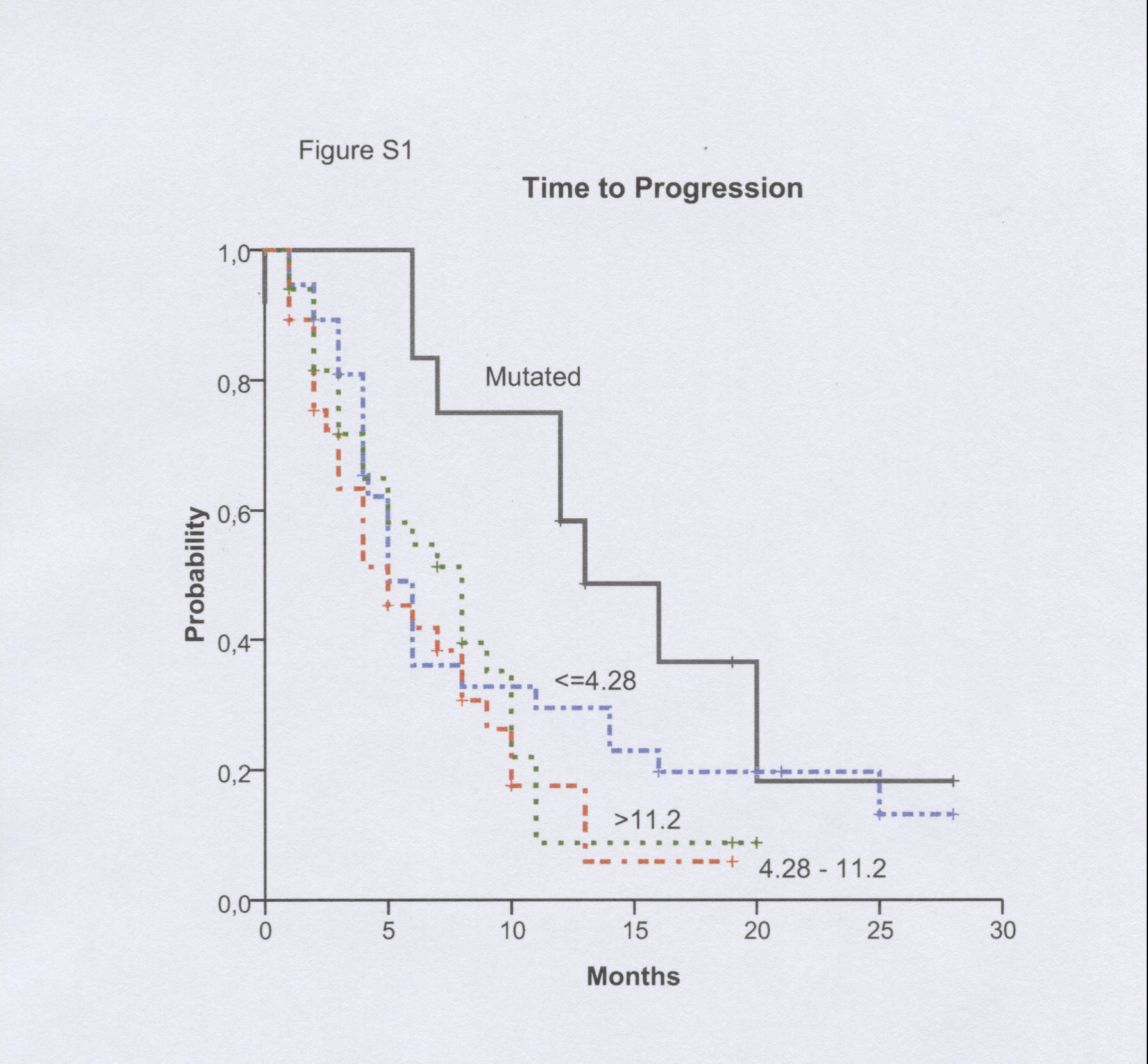

Supplement: Figure S1 — Time to progression according to treatment group. Time to progression was 13 months in the EGFR group, 5 months in the low and intermediate BRCA1 groups, and 8 months in the high BRCA1 group (see Table 2). (8.92 MB TIF) [file pone.0005133.s010.tif]

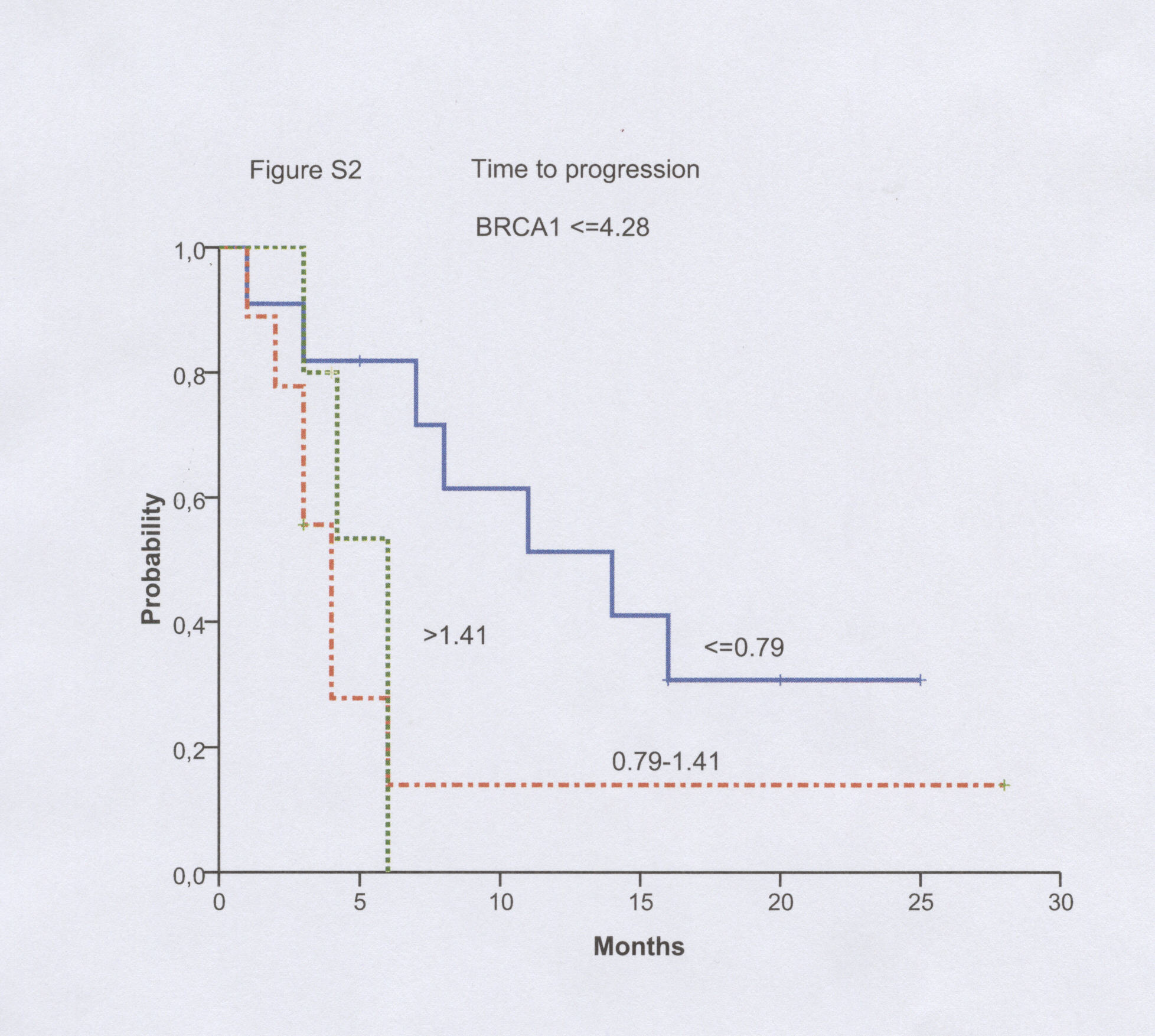

Supplement: Figure S2 — Time to progression for patients in the low BRCA1 group according to RAP 80 expression levels. Time to progression was 14 months for patients with low RAP 80 levels, 4 months for those with intermediate RAP 80 levels, and 6 months for those with high RAP 80 levels (see Table 4). (10.31 MB TIF) [file pone.0005133.s011.tif]
