# Supplementary material for: Customized Treatment in Non-Small-Cell Lung Cancer Based on EGFR Mutations and BRCA1 mRNA Expression
Source: PLoS One. 2009 May 5;4(5):e5133. doi: 10.1371/journal.pone.0005133 (PMC2673583; doi:10.1371/journal.pone.0005133)
Supplement: Protocol S1 — Trial Protocol (1.12 MB PDF) [file pone.0005133.s012.pdf]

Tratamiento individualizado en función de las mutaciones en EGFR y del nivel de expresión de BRCA1 en pacientes con adenocarcinoma de pulmón avanzado

SPANISH LUNG ADENOCARCINOMA TRIAL

SLAT

GECP

Grupo Español de Cáncer de Pulmón

Spanish Lung Cancer Group

PROMOTOR: Grupo Español de Cáncer de Pulmón (GECP)

FECHA DE INICIO: Abril de 2005

INDICE

INFORMACIÓN GENERAL	4
JUSTIFICACIÓN Y MARCO TEÓRICO	5-8
BIBLIOGRAFIA SOBRE EL TEMA	9-12
OBJETIVOS DEL ESTUDIO	13
CRITERIOS DE INCLUSIÓN	14
CRITERIOS DE EXCLUSIÓN	15
ANALISIS ESTADISTICO DE LA MUESTRA	16
GRUPOS DE ESTUDIO	17-18
REGIMENES DE TRATAMIENTO	19
MEDICACIÓN CONCOMITANTE	20
PERIODO DE DURACIÓN DEL TRATAMIENTO	21
PERIODO DEL ESTUDIO	21
MÉTODO/ ENVIO DE MUESTRAS	22-23
ALGORITMO DE ACTUACIÓN	24
EVALUACIONES DURANTE EL ESTUDIO	25
VISITAS DE SEGUIMIENTO	26--27
REGOGIDA DE DATOS	28
DOCUMENTO DE CONSENTIMIENTO INFORMADO	29
TOXICIDADES Y MODIFICACIÓN DE DOSIS	30-40

APENDICE

HOJA DE INCLUSION **EN EL ESTUDIO (SLAT)** (Anexo 1)	41
CONSENTIMIENTO INFORMADO (Anexo 2)	42
DATOS PARA ENVIO DE MUESTRAS Y CRDs (Anexo 3)	43

CENTRIFUGACIÓN DE LAS MUESTRAS DE SANGRE (Anexo 4) 44

ENVIO DE LAS MUESTRAS (Anexo 5) 45

TOXICIDAD GASTROINTESTINAL (Anexo 6) 46

TOXICIDAD CUTANEA (Anexo 7) 47

PETICIÓN DE MEDICACIÓN COMPASIVA (Anexo 8) 48

Spanish Lung Adenocarcinoma Trial (SLAT)

Estudio de tratamiento individualizado en función de las mutaciones en EGFR y del nivel de expresión de BRCA1 en pacientes con adenocarcinoma de pulmón avanzado

Promotor:

Grupo Español de Cáncer de Pulmón (GECP)

Investigador Principal:

Rafael Rosell

Coordinadores:

Miquel Tarón
Noemí Reguart
Teresa Morán

Centros participantes:

Pendientes de determinar (SLAT)

Diseño del estudio:

Estudio farmacogenómico, prospectivo, predictivo y multicéntrico, en pacientes con adenocarcinoma de pulmón avanzado.

Total de pacientes a incluir:

En la primera fase se incluirán un máximo de 120 pacientes y en la segunda fase un máximo de 2000.

Se calcula aproximadamente del 15% de los pacientes sometidos al cribaje de las mutaciones/ deleciones de EGFR (Receptor del Factor de Crecimiento Epidérmico), serán portadores de dichas mutaciones.

Población de estudio: pacientes afectos de carcinoma de pulmón en estadio avanzado con histología de adenocarcinoma, tumores indiferenciados y BAC**.**

JUSTIFICACIÓN Y MARCO TEÓRICO

En la actualidad, nuevos avances en el conocimiento genético-molecular de los factores condicionantes de respuesta a fármacos esta determinando el manejo terapéutico de cierto tipo de tumores. En el caso del carcinoma de pulmón de célula no pequeña (CPCNP), estudios recientes a nivel molecular han demostrado una clara relación entre la presencia o no de mutaciones en el Receptor del Factor de Crecimiento Epidérmico (EGFR) y la respuesta a fármacos tipo inhibidores de la tirosin-kinasa (erlotinib y gefitinib), principalmente en el subgrupo de pacientes con adenocarcinoma de pulmón. La presencia de dichas mutaciones aumenta la actividad tirosin-kinasa y al mismo tiempo confiere sensibilidad a este tipo de tratamiento. A su vez cada vez hay mas evidencia del papel de genes de reparación del DNA en la respuesta tumoral a agentes citotóxicos. Bajo estas premisas se pretende diseñar un nuevo enfoque de tratamiento individualizado, en pacientes con adenocarcinoma de pulmón, por una parte, mediante la determinación de mutaciones en EGFR y por otra mediante la determinación de los niveles de expresión de BRCA1, ambos como marcadores predictivos de respuesta al tratamiento

The Spanish Lung Adenocarcinoma Trial (SLAT):

- Objetivo principal: en la primera fase del estudio (Fase II) el objetivo principal del estudio será evaluar la respuesta al tratamiento. En la segunda fase se analizará el beneficio en supervivencia y tiempo a la progresión en pacientes con adenocarcinoma de pulmón en estadio avanzado, mediante la individualización del tratamiento a partir de la determinación de mutaciones en EGFR (Receptor del Factor de Crecimiento Epidérmico) y los niveles de expresión de BRCA1 (Breast Cancer 1)

- Hipótesis de trabajo: las mejores respuestas obtenidas con agentes inhibidores de la tirosin-kinasa (gefitinib y erlotinib) en CPCNP son del 15% y su combinación con agentes citotóxicos no añade ningún beneficio en la supervivencia. No obstante, en pacientes con adenocarcinoma de pulmón y mutaciones en EGFR, éste tipo de fármacos se han asociado a un mayor índice de respuestas a (hasta el 90%), mayor tiempo a la progresión (12 meses) y una supervivencia media a los 2 años del 80%. Mediante técnicas de Secuenciación y GenScan se pueden identificar el 15-20% de adenocarcinomas de pulmón que se estima serán portadores de mutaciones en EGFR y se podrían beneficiar de tratamiento con inhibidores de la tirosin-kinasa. Junto con RRM1 y ERCC1, Breast Cancer 1 (BRCA1) forma parte del complejo de genes de reparación del DNA, siendo este último el gen más consolidado como marcador molecular. Los niveles de expresión de BRCA1 modulan la respuesta según el tipo de fármacos (resistencia a cisplatino y sensibilidad a agentes antimicrotúbulo). Según nuestra hipótesis de trabajo, en aquellos pacientes sin mutaciones en EGFR, los niveles de expresión de BRCA1 podrían ayudar a individualizar el tratamiento con quimioterapia. Los pacientes con niveles bajos de BRCA1 se beneficiarían de realizar

tratamiento con cisplatino en combinación con gemcitabina, un agente neutral para BRCA1, mientras que aquellos pacientes con niveles de expresión altos de BRCA1 obtendrían un mayor beneficio con agentes antimicrotúbulo. Los pacientes con niveles intermedios para BRCA1 se beneficiarían de realizar tratamiento combinado de Cisplatino con un agente antimicrotúbulo. El diagrama asociado (Figura 2) muestra el concepto de tratamiento individualizado propuesto para este estudio.

- Metodología: todos los servicios que deseen de Oncología Médica de España, podrán participar en el estudio. Solo podrán incluirse pacientes con histología demostrada de adenocarcinoma de pulmón, carcinomas indiferenciados y BAC. La extracción de DNA y mRNA necesarios para el estudio, se obtendrán a partir de las muestras tumorales incluidas en parafina. Las mutaciones en EGFR se analizaran en tejido mediante técnicas de secuenciación y GenScan de los exones 18, 19 y 20. Los niveles de expresión de BRCA1 se analizaran en tejido mediante técnicas de PCR cuantitativa a tiempo real. Los pacientes serán correctamente incluidos en la base de datos para el estudio y la data manager se encargará de dar los resultados a cada uno de los investigadores en un periodo de 5 a 7 días después de la recepción de las muestras. Los pacientes con mutaciones en EGFR realizaran tratamiento oral con un erlotinib (Tarceva, se facilitara a los investigadores a través del estudio TARGET en primera línea en aquellos que formen parte del estudio, mientras que aquellos que no formen parte del mismo deberán solicitar la medicación como uso compasivo) y deberán enviar una muestra de sangre basal previa al inicio del tratamiento. Para los pacientes sin mutaciones en EGFR se determinaran los niveles de expresión de BRCA1. Los pacientes con niveles bajos de BRCA1 realizaran cisplatino+gemcitabina; los pacientes con niveles intermedios de BRCA1 recibirán tratamiento con cisplatino mas docetaxel Finalmente los pacientes con niveles mas elevados de BRCA1 realizaran tratamiento con docetaxel.

- Resultados previstos: se espera que la respuesta en el estudio fase II para los pacientes que presenten la mutación sea superior al 70% y aquellos pacientes tratados según niveles de BRCA1 superen el 30%. La supervivencia media de los pacientes con carcinoma de pulmón de célula no pequeña, incluyendo aquellos con adenocarcinoma de pulmón, se mantiene entre 8 y 9 meses. En los pacientes con adenocarcinoma portadores de mutaciones en EGFR (estimados de 20%) la supervivencia media puede alcanzar los 36 meses con respuestas clínicas del 90%. Así mismo, en los pacientes sin mutaciones en EGFR, los niveles de expresión de BRCA1 pueden ser utilizados como marcador para incrementar la supervivencia en el grupo no mutado hasta 18 meses en el subgrupo de pacientes con niveles bajos de expresión (30%). Los otros dos subgrupos de pacientes con niveles intermedios (25%) y altos de expresión (25%), también se beneficiarían del tratamiento individualizado con supervivencias medias de 15 y 12 meses respectivamente. (Figura 1).

Figura 1: Supervivencias estimadas por subgrupos de tratamiento

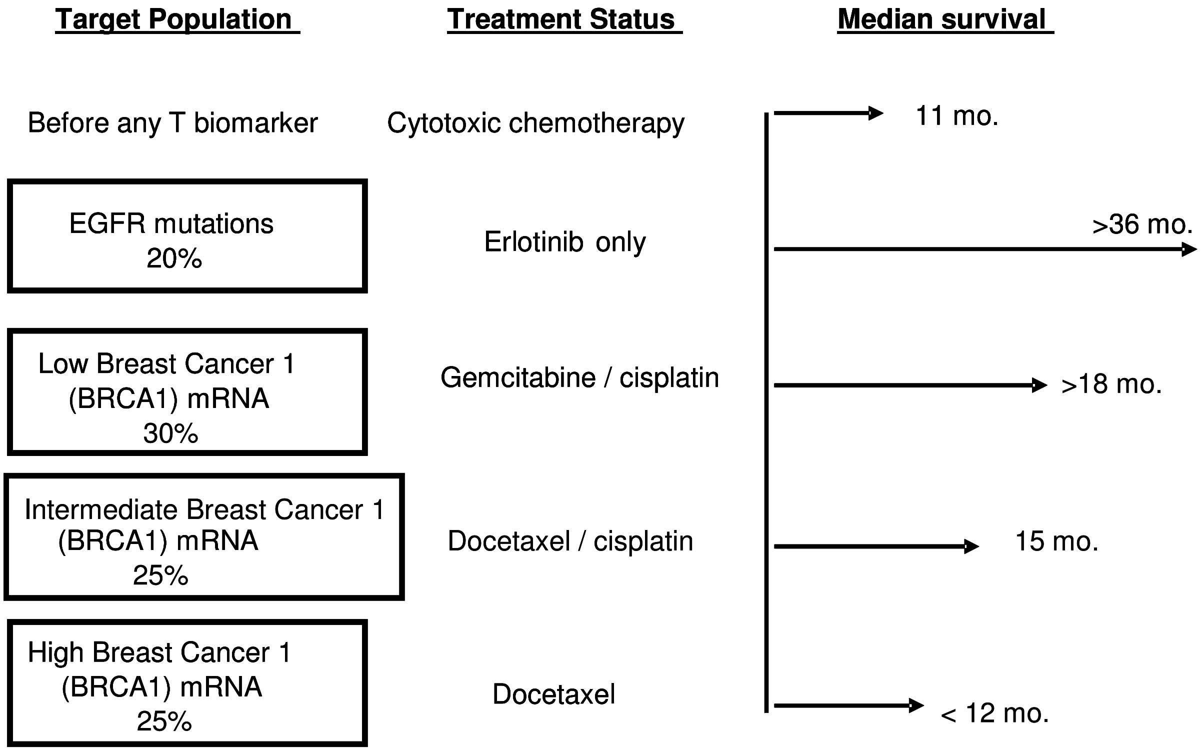

Figura 2: Algoritmo de tratamiento en adenocarcinomas según las determinaciones de EGFR y BRCA1

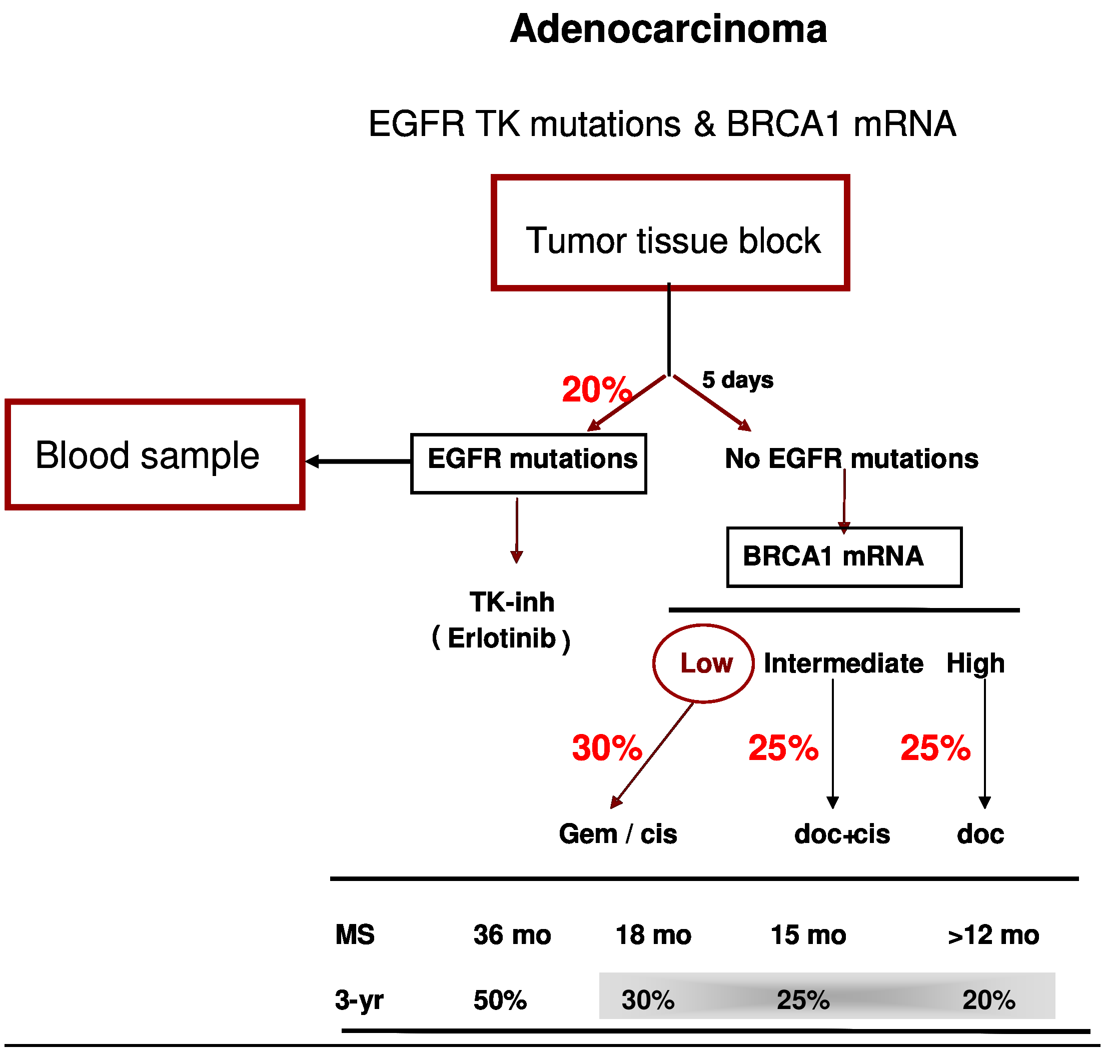

BACKGROUND

Lung cancer is the most common cancer in the world today (12.3 % of all new cases). Non-small-cell lung cancer (NSCLC) accounts for more than 80% of all lung cancers, with an estimated 1.2 million new cases and 1.1 million deaths worldwide in 2001 (17.8% of all cancer deaths), and is the leading cause of cancer-related mortality in both men and women (31% and 25% respectively).[1] Lung cancer currently accounts for one third of all cancer-related deaths in the European Union, or 341,800 lives annually.

Non-small-cell lung cancer is classified in three histologic types: adenocarcinoma, squamous cell carcinoma and large cell carcinoma. Adenocarcinoma is the most frequent histologic type of NSCLC in both sexes in many parts of the world. Previously, squamous cell carcinoma was the predominant form of NSCLC, but in the last few decades it has been replaced by adenocarcinoma. The global rise in lung cancer incidence, together with the fact that the overall 5-year survival of patients with this disease is less than 15%, underscores the failure of lung cancer management.

At present, a reliable biomarker assay for assessing the treatment response of NSCLC patients is not available. Consequently, physicians have been left with uncertainty as to which form of treatment to use first and how best to combine different types of therapy. Two courses of chemotherapy are traditionally given before imaging is done to evaluate the treatment response of NSCLC patients. In patients with advanced NSCLC, treatment with platinum doublets reduces tumor size by 50% or more in approximately 20% of patients and prolongs median survival from approximately 4.8 months[2] (without treatment) to about 11 months[3]. Therefore, the incorporation of biomarkers into treatment plans for individual patients remained to be determined.

Lung cancer is characterized by an enormous accumulation of different genetic or epigenetic alterations (such as inactivation of TSG or activations of oncogenes) that let the cells growth and multiple without any control. Deregulation of protein kinase activity is common in different malignances[4,5]. Some of such therapies have been introduced into the clinical management of advanced NSCLC. This is the case of Epidermal Growth Factor Receptor (EGFR) antagonists (gefitinib and erlotinib). Gefitinib (Iressa, ZD1839) and Erlotinib (Tarceva, OSI-774) are both small-molecules tyrosine kinase (TK) inhibitors with evidence of anti-tumor activity in vitro and in vivo in a range of cancers including NSCLC. They inhibit the protein kinase activity of Epidermal Growth Factor Receptor (EGFR) which is over expressed in many epithelial cancers including

---

[1] Jemal A, Murria T, Ward E, et al. Cancer statistics, 2005. Ca Cancer J Clin 2005;55:10-30
[2] Ranson M, Davidson N, Nicolson M, et al. Randomized trial of paclitaxel plus supportive care versus supportive care for patients with advanced non-small-cell lung cancer. J Natl Cancer Inst 2000;92:1074-80
[3] Fossella F, Pereira JR, van Pawel J, et al Randomized, multinational, phase III study of docetaxel plus platinum combination versus vinorelbine plus cisplatin for advanced non-small-cell lung cancer: The TAX 326 Study Group. J Clin Oncol 2003;21:3016-24
[4] Davies H, Bignell GR, Cox C, et al. Mutations of the BRAF gene in human cancer. Nature 2002; 417:949-54.
[5] Samuels Y, Wang Z, Bardelli A, et al. High frequency of mutations of the PIK3CA gene in human cancers. Science 2004;304:554.

NSCLC[6]. One interesting observation about these new drugs is the significant variability in the response rate. Only 10% to 19% of patients with chemotherapy-refractory advanced NSCLC show a response to this new drug in multi-institutional phase II trials.

Recently reports of EGFR mutations and its role in responsiveness in NSCLC patients have been reported[7,8,9,10,11] . An unprecedented long-term survival is being observed in patients harbouring EGFR mutations and treated with tirosin-kinase inhibitors. In this group of patients with EGFR mutations, the response rate can reach 94% [12]. In a second study, carried out in gefitinib-treated Spanish patients, the time to progression reported for the first time was 12 months in those with EGFR mutations, in contrast to 3.6 months for those without mutations[13]. This prolonged time to progression represents a new landmark in comparison to the less than 3 months for time to progression in second-line treatment, either to chemotherapy drugs such as docetaxel or pemetrexed, or to EGFR inhibitors, such as gefitinib or erlotinib. Those mutations have been found in the tyrosine kinase catalytic domain of EGFR gene, where gefitinib races with adenosine triphosphate (ATP) to binding to the protein. All mutations are located within the exons 18, 19 and 21 of the EGFR kinase domain. Interestingly, those mutations are more frequently found in the adenocarcinoma than in those with other histologies.

Data from several laboratories suggests that ERCC1 could be a useful molecular marker for nucleotide excision repair (NER) activity in that higher ERCC1 mRNA levels have been associated with shorter survival to cisplatin-based chemotherapy. The first report on ERCC1 in NSCLC was published in 2002 in advanced NSCLC patients treated with gemcitabine/cisplatin[14]. Median survival was 5 months for those with high ERCC1 levels, in contrast with 15 months for those with low levels. Later on,

---

[6] Cohen MH, Williams GA, Sridhara R, et al. United States Food and Drug Administration Drug Approval summary: Gefitinib (ZD1839) tablets . Clin Cancer Res 2004;10:1212-8

[7] Lynch TJ, Bell DW, Sordilla R, et al. Activating mutations in the epidermal growth factor receptor expression as a predictive factor for response to gefitinib (”Iressa”, ZD1839) in non-small-cell lung cancer. New Eng J Med 2004;350:2129-39

[8] Paez JG, Jänne PA, Lee JC, et al. EGFR mutations in lung cancer: correlation with clinical response to gefitinib therapy. Science 2004;304:1497-500

[9] Pao W, Miller V, Zakowski M, et al. EGF receptor gene mutations are common in lung cancers from "never smokers" and are associated with sensitivity of tumors to gefitinib and erlotinib *Proc Natl Acad Sci U S A. 2004 Sep 7;101(36):13306-11*

[10] Kim KS, Jeong JY, Kim YC, et al. Predictors of the response to gefitinib in refractory non-small cell lung cancer. Clin Cancer Res. 2005 Mar 15;11(6):2244-51.

[11] Yang SH, Mechanic LE, Yang P, et al. .Mutations in the tyrosine kinase domain of the epidermal growth factor receptor in non-small cell lung cancer.. Clin Cancer Res. 2005 Mar 15;11(6):2106-10.

[12] Miguel Taron, Yukito Ichinose, Rafael Rosell, et al. Activating Mutations in the Tyrosine Kinase Domain of the Epidermal Growth Factor Receptor are Associated with Gefitinib Response in Chemorefractory Lung Adenocarcinomas. *Clin Cancer Res.* (Accepted, to be published 2005).

[13] Hernan Cortes-Funes, Carlos Gomez, Rafael Rosell, et al. Epidermal Growth Factor Receptor Activating Mutations in Spanish Gefitinib-Treated Non-Small-Cell Lung Cancer Patients on behalf of the Spanish Lung Cancer Group. *Annals of Oncology* (Accepted, to be published 2005).

[14] Lord RV, Brabender J, Gandara D, et al. Low ERCC1 expression correlates with prolonged survival after cisplatin plus gemcitabine chemotherapy in non-small cell lung cancer. *Clin Cancer Res. 2002 Jul;8(7):2286-91.*

ribonucleotide reductase (RRM1) was identified as a molecular marker in gemcitabine/cisplatin-treated NSCLC patients, also with striking differences in survival[15].

In 2004, close correlation between RRM1 and ERCC1 was observed, and also with BRCA1[16]. Among the three transcripts (ERCC1, RRM1 and BRCA1), the latter surfaced as the most robust molecular marker.

BRCA1 (Breast Cancer 1) is involved in TC-NER (Transcription-Coupled Nucleotide Excision Repair). The BRCA1 gene encodes responds to DNA damage participating in cellular pathways responsible for DNA repair, mRNA transcription, cell cycle regulation and protein ubiquitination. Moreover decreased BRCA1 expression is observed in both breast and ovarian cancers[17]. BRCA1 acts as a differential modulator of apoptosis depending on the nature of the cellular insult. In preclinical models, BRCA1 mediates sensitivity to apoptosis induced by antimicrotubule agents such as paclitaxel or vinorelbine but conversely induces resistance to DNA-damaging agents such as cisplatin or etoposide[18] by activating the S-phase and G2/M-phase DNA-damage checkpoints. Therefore, the loss of BRCA1 function selects cancer cell death after DNA-damaging treatments.

This year, low levels of ERCC1 mRNA are observed more frequently in lung adenocarcinoma than in other NSCLC histologies, such as squamous cell carcinoma. Patients with low ERCC1 or BRCA1 mRNA levels are potentially more sensitive to cisplatin-based chemotherapy. In summary, lung adenocarcinomas have lower DNA repair capacity, as demonstrated in several studies. Individuals homozygous for the variant methionine of the XRCC3 241 SNP have an increased number of DNA adducts in their leucocytes. In addition, lung adenocarcinomas have a higher frequency of the XRCC3 homozygous methionine allele. EGFR mutations are almost exclusively found in lung adenocarcinomas. Therefore, a molecular assay analyzing EGFR mutations and BRCA1 mRNA levels could provide a new therapeutic approach for lung adenocarcinoma. Between 10% and 20% of lung adenocarcinomas can contain EGFR mutations, and the treatment with oral EGFR inhibitors can provide dramatic and durable responses with disappearance of all metastatic lesions. It can be predicted that median survival can triplicate in these patients; instead of less than 1 year it can increase to almost 3 years.

For the vast majority of lung adenocarcinomas without EGFR mutations, BRCA1 mRNA customized chemotherapy can also improve survival, especially in the subgroup of patients (near 30%) with low levels of BRCA1 in whom gemcitabine/cisplatin can expect to double median survival, up to 20 months. Belfast investigators in 2003 fully

---

[15] Rosell R, Scagliotti G, Danenberg KD, et al**.** Transcripts in pretreatment biopsies from a three-arm randomized trial in metastatic non-small-cell lung cancer. *Oncogene 2003 Jun 5;22(23):3548-53*

[16] Rosell R, Danenberg KD, Alberola V, et al. Ribonucleotide reductase messenger RNA expression and survival in gemcitabine/cisplatin-treated advanced non-small cell lung cancer patients**.**. *Clin Cancer Res. 2004 Feb 15;10(4):1318-25*

[17] Kennedy RD, Quinn JE, Johnston PG, et al. BRCA1: mechanisms of inactivation and implications for management of patients. *Lancet. 2002 Sep 28;360(9338):1007-14*

[18] Quinn JE, Kennedy RD, Mullan PB, et al. BRCA1 functions as a differential modulator of chemotherapy-induced apoptosis. *Cancer Res. 2003 Oct 1;63(19):6221-8.*

documented that BRCA1 displays a differential effect in chemosensitivity[18]. Low levels increase sensitivity to cisplatin, while high levels confer cisplatin resistance and highly enhance sensitivity to antimicrotubule drugs, such as taxanes and vinca alkaloids. Although gemcitabine is a BRCA1 neutral drug, the fact that the levels of RRM1 are closely related to those of BRCA1 precludes the use of gemcitabine in patients with the highest BRCA1 levels. This is the main core of the hypothesis of the present study

OBJETIVOS

Objetivo primario:

En el estudio fase II:

1.- Respuesta tumoral: los criterios de respuesta para las lesiones diana se definen de acuerdo a los criterios RECIST especificados en el siguiente apartado. La respuesta objetiva (respuesta completa o parcial) debe confirmarse con una segunda medición al menos 4 semanas después de la primera medición.

En la segunda fase del estudio será:

1. **Supervivencia:** en el subgrupo de pacientes con mutaciones EGFR tratados con Erlotinib determinada desde el momento de randomización hasta la fecha de muerte del paciente. En caso de que el paciente haya sido perdido de seguimiento, se considerará la última fecha en que se conoce que el paciente sigue vivo por última vez como fecha para la determinación de supervivencia.

Objetivos secundarios:

En el estudio fase II:

1. **Supervivencia:** en el subgrupo de pacientes con mutaciones EGFR tratados con Erlotinib determinada desde el momento de randomización hasta la fecha de muerte del paciente. En caso de que el paciente haya sido perdido de seguimiento, se considerará la última fecha en que se conoce que el paciente sigue vivo por última vez como fecha para la determinación de supervivencia.

2. **Supervivencia libre de progresión:** medida como el tiempo de recaída desde el momento de la randomización hasta la evidencia de progresión, empleando los criterios RECIST.

En la segunda fase será la Respuesta al tratamiento:

1. **Respuesta tumoral:** los criterios de respuesta para las lesiones diana se definen como sigue (adaptado de los criterios RECIST). La respuesta objetiva (respuesta completa o parcial) debe confirmarse con una segunda medición al menos 4 semanas después de la primera medición.

- **Respuesta completa:** desaparición de todas las lesiones diana.

- **Respuesta parcial:** al menos una disminución del 30% en la suma de los diámetros mayores de la(s) lesión/ lesiones diana, tomando como referencia la suma de los diámetros mayores basales.

- **Enfermedad estable:** respuesta insuficiente para clasificarla como respuesta parcial o respuesta completa e incremento insuficiente para clasificarlo como enfermedad progresiva, tomando como referencia la suma de los diámetros mayores basales desde el inicio del tratamiento.
- **Enfermedad en progresión:** al menos un incremento del 20% en la suma de los diámetros mayores de las lesiones diana, tomado como referencia la suma de los diámetros mayores desde el inicio del tratamiento; o bien la aparición de una o más lesiones nuevas

2. **Supervivencia libre de progresión:** medida como el tiempo de recaída desde el momento de la randomización hasta la evidencia de progresión, empleando los criterios RECIST.

3. **Tiempo de duración de respuesta:** determinado desde la fecha en que se objetiva la respuesta tumoral hasta que aparece la progresión.

CRITERIOS DE INCLUSIÓN

- Edad igual o mayor a 18 años.
- Los pacientes deben tener el diagnóstico histológicamente confirmado de **adenocarcinoma** de pulmón.
- Se aceptan casos de **carcinomas indiferenciados y BAC**.
- Sólo se incluirán pacientes con **enfermedad avanzada definida como estadio IV o IIIB con/ sin derrame pleural.**
- Debe existir **muestra tumoral disponible**.
- Debe existir **lesión medible**.
- Karnofsky igual o superior a 60% **(ECOG≥2)**
- Los pacientes no deben haber recibido tratamiento previo con quimioterapia u otros agentes para enfermedad diseminada. Se permite, por tanto, quimioterapia adyuvante/ neoadyuvante, en el supuesto de que el diagnóstico inicial del paciente fuese de enfermedad limitada, así como radioterapia torácica adyuvante/ neoadyuvante, en pacientes con diagnóstico inicial de enfermedad limitada.
- Se permite la inclusión de pacientes con enfermedad cerebral sin limitaciones de tiempo desde la irradiación holocraneal ni tratamiento antiedema complementario.
- Los pacientes deben tener una adecuada función renal, hepática y hematológica.
- El paciente deberá firmar un consentimiento informado previo a la inclusión en el estudio que permita: tratamiento dentro del ensayo y que presupone consentimiento para análisis de muestras biológicas de tumor y de sangre (las muestras de sangre solo para aquellos pacientes positivos para mutaciones en EGFR).
- Los pacientes con potencial reproductivo deberán asegurar correcto método anticonceptivo
- Los pacientes deben estar disponibles para el seguimiento clínico.

CRITERIOS DE EXCLUSIÓN

- Pacientes que hayan recibido algún agente en investigación en los 21 días previos a su inclusión en el estudio.
- Pacientes que hayan recibido tratamiento previo con ningún agente anti-Receptor del Factor de Crecimiento Epidérmico.
- Comorbilidad severa asociada.

ANÁLISIS ESTADÍSTICO Y TAMAÑO DE LA MUESTRA

En el estudio fase II, en el que se incluirán pacientes en dos brazos de tratamiento según presenten o no la mutación del gen EGFR, para el cálculo del tamaño muestral se utiliza el modelo de Ghean. Éste se realizará con las siguientes presunciones; estudio multicéntrico, no-aleatorizado cuyo objetivo principal es estimar la proporción de pacientes que responden en el grupo de pacientes que presenten mutación y el grupo de pacientes tratados en función del nivel de BRCA1, se fija una eficacia mínima del 20%, el estudio se realizará en dos fases con un error de tipo I del 5%, error de tipo II del 10% (1-ß=90%) y precisión de 5% (ε=0,05). En una primera etapa se incluirán un total de 11 pacientes y en una segunda fase se aplicará el siguiente algoritmo:

1.- Parar estudio por elevada eficacia si ≥6 respuesta
2.- 1 respuesta se incrementará en 60 pacientes la muestra
3.- 2 respuestas se incrementará en 77 pacientes la muestra
4.- 3 respuestas se incrementará en 87 pacientes la muestra
5.- 4 respuestas se incrementará en 90 pacientes la muestra
6.- >4 se incrementará la muestra hasta llegar a 90 pacientes

Según este algoritmo se necesitaran como máximo 100 pacientes evaluables teniendo en cuenta un 10% de posibles pérdidas de seguimiento.

En caso de que en alguno de los esquemas propuestos para el Fase II (mutación EGFR, niveles de BRCA1) alcanzara un total de respuestas superior a 6 entre los primeros 11 pacientes, se procederá al cierre de dicho grupo de tratamiento por elevada eficacia del fármaco.

Una vez obtenidos los resultados de la primera etapa del estudio (Fase II) se procederá al cálculo del tamaño muestral para la segunda etapa del estudio (Fase III) en función de los resultados obtenidos y lo publicado respecto al parámetro mediana de supervivencia y tiempo a la progresión.

Para la viabilidad del estudio y por la experiencia del SLCG se estima que el tamaño muestral máximo que podrá ser incluido en la segunda etapa no debe superar los 2000 pacientes reclutados en un período de 2 años.

El análisis estadístico se realizará en dos etapas. La primera será por intención de tratamiento. En dicha fase se incluirán todos los pacientes. En una segunda fase solo se analizarán aquellos pacientes válidos para el análisis estadístico. Serán pacientes no validos para el estudio todos aquellos que después de haber dado su consentimiento informado se nieguen a ser posteriormente tratados, los que no realicen el tratamiento adjudicado según el protocolo y aquellos en los que no haya suficiente material tumoral para realizar el estudio genético.

El objetivo primario de este estudio es comparar las características clínicas, proporciones de respuesta y supervivencia en pacientes con y sin mutaciones en EGFR. Las diferencias entre las proporciones de respuesta y las características clínicas entre los pacientes con y sin mutaciones serán analizados mediante la prueba de Chi-cuadrado, la prueba exacta de Fisher, y la prueba T de Student o la prueba equivalente no paramétrica U de Mann-Whitney. La distribución normal de las variables continuas se evaluará mediante la prueba de Kolmogorov-Smirnov para una muestra. El tiempo de supervivencia será calculado desde el inicio del tratamiento con erlotinib en aquellos pacientes con mutaciones, o desde el inicio de tratamiento con quimioterapia, asignada según los niveles de BRCA1, en caso de que el paciente no presente mutaciones en EGFR.

El Hazard ratio será calculado mediante el modelo univariable de COX y la comparación entre las distintas curvas Kaplan-Meier se realizará mediante la prueba de contraste Log rango**.** Los modelos de regresión logística analizarán la probabilidad de respuesta para cada covariable en los distintos subgrupos después de realizar un ajuste por factores (edad, sexo y historia de tabaquismo). En el análisis de la relación mutación EGFR-respuesta, se utilizarán distintas técnicas para evaluar la consistencia de los resultados, las cuales incluirán análisis crudos univariantes y análisis ajustados por factores de interés. El programa SPSS 11.5 será utilizado para realizar el análisis estadístico.

Con el objetivo de realizar una fácil evaluación del efecto de los niveles de expresión de BRCA1 mRNA en el grupo de pacientes sin mutaciones en EGFR, se dividirán los niveles de expresión en quartiles. Se calcularán en cada quartíl, los coeficientes de variación de los valores de BRCA1 entre pacientes para así valorar similitudes entre los mismos. Todas las pruebas realizadas fueron de dos colas, con un poder estadístico del 80% y un nivel de significación fijado al 5%. Las pruebas fueron significativas a nivel 5% exceptuando las comparaciones múltiples donde se aplicará la corrección de Bonferroni.

GRUPOS DE ESTUDIO

Grupo 1

El grupo de pacientes portadores de mutación en EGFR recibirán tratamiento con **Erlotinib** (será facilitado al investigador a través del estudio TARGET en 1ª línea o bien solicitado como uso compasivo en caso de no participar del estudio TARGET), un inhibidor oral selectivo de la tirosín kinasa del Receptor del Factor de Crecimiento Epidérmico

Tras presentarse una enmienda al protocolo TARGET (del cual forman parte varios centros a nivel nacional) y con el objeto de aumentar el tamaño muestral, se incluyó un cuarto estrato de tratamiento referido a pacientes con diagnóstico histológico de adenocarcinoma, en estadio avanzado con presencia de mutación/deleción de los exones 19-21 del EGFR, a los cuales se administraría Erlotinib en primera línea de tratamiento

Roche facilitará Erlotinib (Tarceva) a aquellos investigadores que formen parte del estudio TARGET y cuyos pacientes que cumplan los criterios de inclusión y presenten mutaciones en EGFR. En el caso de que el centro no forme parte del estudio TARGET, el investigador deberá conseguir el medicamento por uso compasivo. Para agilizar la solicitud por uso compasivo, hemos facilitado la dirección de correo electrónico (Anexo 6) de la cual los investigadores podrán recibir un dossier de solicitud de medicación compasiva con las bases científicas que apoyan el tratamiento con Erlotinib. Esto permitirá a los investigadores obtener la aprobación por el Ministerio en el plazo de una semana.

Grupo 2

El resto de los pacientes no portadores de la mutación del Receptor del Factor de Crecimiento Epidérmico (**Grupo 2**) recibirán tratamiento con **quimioterapia individualizada en función de los niveles de BRCA 1 m RNA. En función de dichos niveles se establecerá tres subgrupos diferentes de tratamiento:**

- Subgrupo A con niveles bajos de BRCA1 mRNA recibirán tratamiento con **gemcitabina/ cisplatino**

- Subgrupo B con niveles intermedios de BRCA1 mRNA: recibirán tratamiento con **docetaxel/ cisplatino**

- Subgrupo C con niveles altos de BRCA1 mRNA: recibirán tratamiento con **docetaxel**

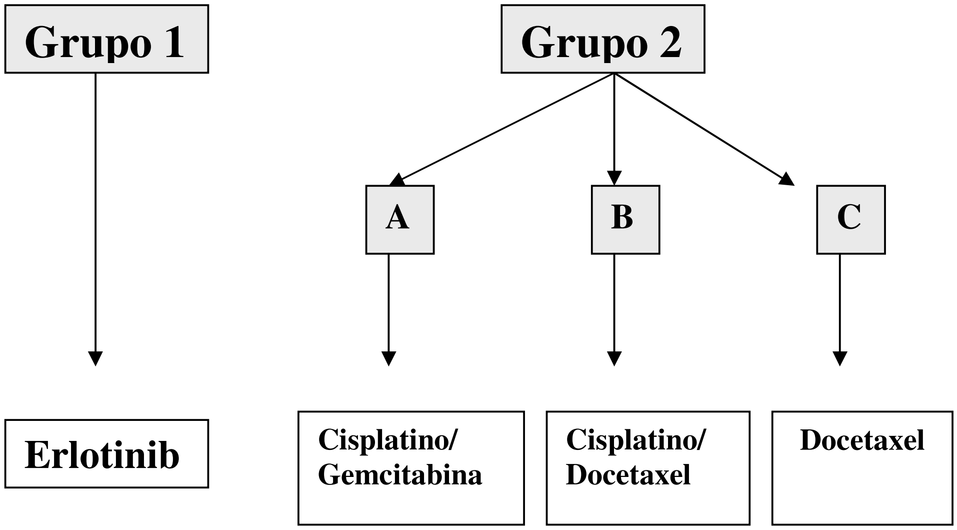
Grupo 1
Grupo 2
A
B
C
Erlotinib
Cisplatino/
Gemcitabina
Cisplatino/
Docetaxel
Docetaxel

REGIMENES DE TRATAMIENTO:

Pacientes del grupo 1:

Los pacientes incluidos en el Grupo 1 portadores de la mutación de Receptor del Factor de Crecimiento Epidérmico, recibirán tratamiento vía oral con **Erlotinib 150 mg** (una dosis diaria) inicialmente durante 21 días y después de forma continua hasta la progresión**.**

Pacientes del grupo 2:

Los pacientes del Grupo 2 recibirán tratamiento con quimioterapia individualizada en función de los niveles de BRCA1 m RNA:

- **A** Los pacientes con bajos niveles de expresión de BRCA1 en el tejido tumoral recibirán **gemcitabina 1250 mg/ m2 ev días 1 y 8, y cisplatino 75 mg/ m2 ev día 1.** Ciclos cada 21 días. Total de ciclos: 6, pudiéndose prolongar a 8 ciclos a criterio del investigador.

- **B** Los pacientes con niveles intermedios de expresión de BRCA1 recibirán **docetaxel 75 mg/ m2 y cisplatino 75mg/ m2,** ambos administrados el día 1, cada 21 días en un total de 6 ciclos, pudiéndose prolongar a 8 ciclos a criterio del investigador.

- **C** Los pacientes con niveles altos de expresión de BRCA1 recibirán **docetaxel 75 mg/ m2**, **día 1**, cada 21 días, total de ciclos: 6, pudiéndose prolongar a 8 ciclos a criterio del investigador

MEDICACIÓN CONCOMITANTE:

Pacientes del grupo 1:

No existe restricción durante la inclusión o durante el período de tratamiento con ninguna medicación concomitante que los pacientes puedan estar tomando, en ninguno de los grupos de tratamiento.

Dada la existencia de potencial interacción con anticoagulantes en caso de que el paciente esté recibiendo tratamiento con dicumarínicos deberá realizarse un control más estricto por parte del investigador.

Pacientes del grupo 2:

- **Premedicación antiemética**.

  Se recomienda la administración profiláctica de tratamiento antiemético agresivo. El tipo de tratamiento quedará a criterio del investigador.

- **Premedicación con corticoides.**

  Es recomendable la administración del siguiente régimen de premedicación a todos los pacientes tratados con docetaxel, con el objeto de prevenir la presentación de una reacción de hipersensibilidad y para reducir y/o retrasar la presentación de toxicidad cutánea y de retención de líquidos relacionadas con docetaxel.

  Dexametasona 8 mg (ó equivalente: Metilprednisolona 40 mg; Prednisona ó prednisolona 50 mg) por vía oral: 12 h previas a la quimioterapia, 3 h previas a la quimioterapia, una hora antes de la infusión de docetaxel, 12 h después de la quimioterapia, 24 h tras la quimioterapia, 36 h tras la quimioterapia.

- **Pauta de hidratación para cisplatino:**

  Se seguirá la pauta de hiperhidratación y diuresis forzada para la administración de cisplatino propia de cada Centro.

PERIODO DE DURACIÓN DEL TRATAMIENTO:

Pacientes del grupo 1:

Los pacientes del grupo 1 continuarán tratamiento con **Erlotinib** hasta la progresión, hasta que aparezca una toxicidad inaceptable o hasta que ocurra un efecto adverso grave, siempre que el paciente rechace seguir participando en el estudio o a criterio del investigador que crea conveniente la salida del paciente del estudio.

Pacientes del grupo 2:

Los pacientes del grupo 2 realizarán tratamiento de **quimioterapia** hasta completar los seis ciclos de quimioterapia (o bien 8 a criterio del investigador).

PERIODO DEL ESTUDIO:

La fecha planeada para el inicio del estudio es Mayo de 2005 y se considera que en un período de 2 años puede estar completado el reclutamiento.

El período de seguimiento se calcula de 24 meses desde la inclusión del último paciente.

MÉTODO PARA ENVÍO DE MUESTRAS

1. **Es obligado que exista suficiente tejido tumoral** para realizar el análisis MOLECULAR a fin de poder determinar las mutaciones del Receptor del Factor de Crecimiento Epidérmico.

2. Tras la **firma del consentimiento informado** por parte del paciente o representante legal, se enviará la **hoja de inclusión** por fax (Anexo 1) del paciente a la datas managers del estudio Esther Carrasco Chaumel/ Mireia Tomás Solá (Anexo 3) que adjudicará un número de inclusión al paciente.

3. En el momento de la inclusión se enviará el bloque de parafina del tejido tumoral del paciente al **Laboratorio de Biología Molecular del Hospital Germans Trias i Pujol** para el análisis de las mutaciones del Receptor del Factor de Crecimiento Epidérmico. El bloque será devuelto a cada centro de referencia tras completar los estudios.

4. En el envío, deberá incluirse una hoja con el **informe anatomopatológico** del centro hospitalario en cuestión (de mayor interés en aquellos casos de carcinomas indiferenciados).

5. Se debe incluir un corte del mismo bloque **parafinado teñido con hematoxilina/ eosina** (esto nos asegura el diagnostico de adenocarcinoma por parte del patólogo de cada centro y permite a su vez una revisión por parte del Servicio de Anatomía Patológica del Hospital Germans Trias i Pujol).

6. **Se enviarán muestras de sangre periférica SOLO para el subgrupo de pacientes con mutaciones positivas para EGFR (20%)**. Una vez el investigador posea el resultado positivo para mutaciones en EGFR, deberá enviar un tubo lila (EDTA/K3) con 10 ml de sangre sin centrifugar y dos tubos "camuflaje" con 10 ml de sangre en cada uno que deberán centrifugarse y transferirse a un tubo limpio (anexo 4, anexo 5). La data manager del estudio se encargará de facilitar los tubos a los centros que posean un paciente positivo para mutaciones de EGFR (en caso de duda ponerse en contacto con la data manager Anexo 3). Los tubos estarán dentro de una caja de protección identificada con el membrete SLADB/SLAT. Los tubos deberán también adjuntar una pegatina de identificación con el código del paciente y fecha de extracción (las pegatinas estarán incluidas dentro del la caja con los tubos para las extracciones). Es muy importante que la sangre sea remitida previo inicio de tratamiento con Erlotinib.

7. El envío de las muestras se realizará a temperatura ambiente y gestionada a través del **servicio de mensajería de MRW** (Anexo 3). **Adjuntar siempre una copia de la hoja de envío con de la muestra de sangre para poder identificar al paciente** (anexo 1).

8. Aproximadamente en el plazo de una semana se remitirá la misma **hoja de inclusión** (anexo 1) vía fax a cada centro, con el grupo de tratamiento asignado (en caso de cualquier duda contactar con la data manager del estudio Anexo 3)

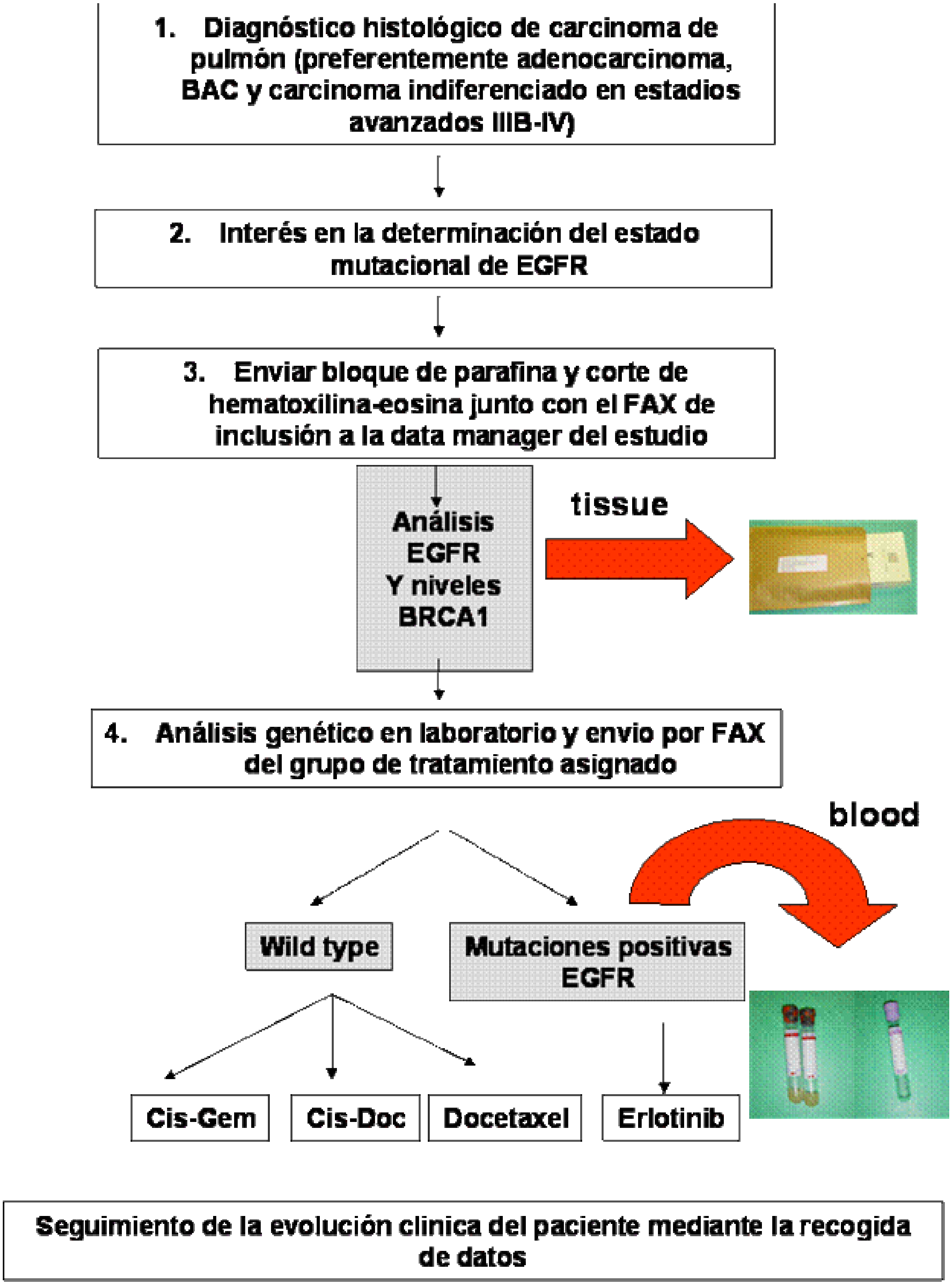
1. Diagnóstico histológico de carcinoma de pulmón (preferentemente adenocarcinoma, BAC y carcinoma indiferenciado en estadios avanzados IIIB-IV)
2. Interés en la determinación del estado mutacional de EGFR
3. Enviar bloque de parafina y corte de hematoxilina-eosina junto con el FAX de inclusión a la data manager del estudio
Análisis EGFR Y niveles BRCA1
tissue
4. Análisis genético en laboratorio y envio por FAX del grupo de tratamiento asignado
blood
Wild type
Mutaciones positivas EGFR
Cis-Gem
Cis-Doc
Docetaxel
Erlotinib
Seguimiento de la evolución clinica del paciente mediante la recogida de datos

EVALUACIONES DURANTE EL ESTUDIO:

- Para la inclusión del paciente en el estudio deberá evaluarse la historia clínica y la exploración física en las 3 o 4 semanas previas a la inclusión. Deberá realizarse en ese mismo plazo una analítica general de rutina que incluya hematología, coagulación y bioquímica con función hepática, renal, e ionograma.

- La historia clínica deberá recoger la existencia previa de **patología cutánea**.

- En el periodo de 3- 4 semanas previos a la inclusión deberá tenerse una **valoración por TAC** de todas las lesiones medibles en los dos grupos de tratamiento.

- **Aunque no es obligatorio los centros que tengan disponibilidad para la realización de TAC-PET, sería recomendable su realización dentro de los estudios basales principalmente en aquellos pacientes que vayan a realizar tratamiento con erlotinib.** En aquellos centros en los que no se disponga de facilidad para realizar una TAC-PET basal el control se realizará mediante TAC u otras pruebas adicionales en caso de lesiones previas o clínica nueva que sugiera la posibilidad de nuevas lesiones no valorables con el TAC.

- En los pacientes del **grupo 1** (portadores de las mutaciones del Receptor del Factor de Crecimiento Epidérmico) en que se haya podido realizar un **TAC-PET** basal, debería realizarse una **primera valoración precoz** en el plazo de las 3 o 4 semanas posteriores al inicio del tratamiento con el inhibidor oral.

- Las pruebas de imagen deberán realizarse cada 2 ó 3 meses en función del calendario de seguimiento de cada centro (no se ha realizado un calendario estricto para las pruebas de valoración, por lo que se aceptarán las propias de cada centro).

VISITAS DE SEGUIMIENTO

El seguimiento de los pacientes del **grupo 1** se realizará cada 2 ó 3 meses mientras dure el tratamiento con erlotinib, es decir hasta la PE, muerte o salida del estudio.

El seguimiento de los pacientes del **grupo 2** tras finalizar el tratamiento con quimioterapia incluirá TAC, así como otras pruebas de imagen adicionales en caso de lesiones previas o sospecha de nuevas lesiones no valorables con el TAC. El período para repetir el TAC de control dependerá del calendario de control de cada centro, pero deberá realizarse cada 3 o 4 meses hasta la PE.

El **grupo 1 y 2**, una vez finalizado el tratamiento, deberán seguir completando un registro de las segundas líneas de tratamiento así como fecha de éxitus, para recoger la supervivencia global.

Parámetro	Pre-Tratamiento (3-4 semanas previas)	Durante el Tratamiento	Fin tratamiento	Seguimiento (cada 3 meses o hasta PE)
**Historia Médica** (recordar historiar patología cutánea)	X	X	X	X
**Exploración física:**				
• Altura	X			
• Peso	X			
• ECOG	X	X	X	X
**Análisis sanguíneo:**				
• Hematología basal	X	*	*	*
• Bioquímica basal	X	*	*	*
• Coagulación	X			
**Envío de muestra sanguínea**	X			
**Envío de muestra de tejido tumoral**	X			
**Valoración de toxicidad**		continuo		
**Radiología:**				
TAC tórax	X	X	X	X
PET-TAC[1]	X	X[2]	X	X
Otras pruebas de valoración	X	X	X	X

** A criterio del centro hospitalario*
[1] opcional realización de PET en función de la disponibilidad del centro hospitalario en los pacientes del grupo 1 (Erlotinib)
[2] se aconseja la realización de una valoración precoz (1er mes de tratamiento) en los pacientes del grupo 1 (Erlotinib) en los que se disponga de PET-TAC

RECOGIDA DE DATOS

- Se ha establecido que la recogida de datos y la introducción en la base de datos para posterior análisis estadístico estará centralizada en el Hospital Universitario Germans Trias i Pujol, donde estará la base de datos y las data managers (Esther Carrasco Chaumel/ Mireia Tomás Solá).

- La inclusión de cada paciente debe ir acompañada de un formulario basal (CRF) y los datos de seguimiento deberán irse actualizando periódicamente mediante un formulario específico que deberá remitirse por fax. Estos formularios forman parte del Cuaderno de Recogida de Datos o CRFs de los cuales dispondrán todos los investigadores que participen en el estudio

- Existe una formulario basal común y formularios específicos de valoración inicial seguimiento y salida para cada grupo de tratamiento (grupo 1 y grupo 2):
  - **Grupo 1:**
    - Formulario basal (que es común para el grupo 1 y 2) donde se recogen los datos basales de la enfermedad.
    - Formulario de primera valoración donde se valorará las primeras pruebas de imagen, respuesta tumoral y toxicidad cutánea y gastrointestinal (las únicas solicitadas en este grupo de tratamiento) tras el inicio de Erlotinib.
    - Formulario de seguimiento en tratamiento con Erlotinib que se realizará en cada visita de valoración mientras el paciente este en tratamiento con Erlotinib. Deberá remitirse la hoja de seguimiento por Fax con la fecha de visita. En éste se reportará el estado de la enfermedad, la situación del paciente (vivo, muerto), ECOG y toxicidades.
    - Formulario de salida de protocolo en donde se solicita la fecha de salida, el estado final de la enfermedad, el estado del paciente (vivo, muerto) para calcular OS y segunda línea en caso de progresión.
    - Formulario de muerte
  - **Grupo 2:**
    - Formulario basal
    - Formulario de valoración inicial tras 3 o 4 ciclos de tratamiento
    - Formulario de valoración final tras 6 o 8 ciclos de tratamiento
    - Formulario de salida de protocolo
    - Formulario de seguimiento en este caso una vez finalizado el tratamiento
    - Formulario de muerte

*** Se ha intentado que estos formularios sean lo más simplificados posibles para facilitar el trabajo de la recogida de datos.**

DOCUMENTO DEL CONSENTIMIENTO INFORMADO:

Existe un documento de consentimiento informado que el paciente/ representante deberá firmar previa inclusión en el ensayo y que presupone: consentimiento de paciente para participar en el estudio, para análisis de la muestra de su tumor y para análisis de las muestras de sangre (anexo 4).

TOXICIDADES Y MODIFICACIONES DE DOSIS:

PACIENTES DEL GRUPO 1 EN TRATAMIENTO CON ERLOTINIB

Los datos de toxicidad recogidos en los estudios clínicos iniciales muestran que los efectos adversos más frecuentes durante el tratamiento con erlotinib administrado en monoterapia son la afectación cutánea en forma de rash, diarrea, nauseas, astenia, afectación de la mucosa oral con estomatitis, vómito y cefalea. También se han descrito elevaciones transitorias de transaminasas.

Toxicidades descritas

- **Toxicidad sobre piel y mucosas:**

En los estudios fase II de tratamiento con erlotinib en monoterapia las manifestaciones cutáneas fueron frecuentes e incluyeron diferentes tipos de rash, la mayoría inespecíficos (61%) o acneiforme (13%) que cursó con prurito, xerosis cutánea, eritema y alopecia. La media de recuperación de la toxicidad cutánea fue de 8 días. La severidad de la afectación cutánea fue variable y disminuyó después de 4 semanas de tratamiento.

- **Toxicidad gastrointestinal:**

La diarrea, náuseas y vómitos habitualmente son habitualmente intermitentes durante el tratamiento con erlotinib y no suelen requerir reducción de dosis. En los estudio fase II la mediana de aparición de el primer episodio de diarrea es de 9 días. La diarrea fue leve o moderada y se controló correctamente con loperamida. Complicaciones potenciales de la diarrea son deshidratación, hipotensión e insuficiencia renal.

- **Toxicidad pulmonar:**

La mayoría de los datos relativos a seguridad del tratamiento con erlotinib se han obtenido de pacientes afectos de carcinoma de pulmón, en los que existe un elevado número de eventos respiratorios relacionados con la propia enfermedad. Con menor frecuencia se han descrito eventos relacionados con el tratamiento, como la neumonitis intersticial pulmonar y la disnea. La mayoría de estos eventos relacionados con el tratamiento suponen un desenlace fatal para el paciente.

- **Toxicidad hematológica:**

De forma global, no se ha observado toxicidad hematológica en pacientes que reciben erlotinib en monoterapia.

Trastornos de la coagulación en forma de un alargamiento del tiempo de protrombina han sido descritos en pacientes con tratamiento concomitante con anticoagulantes dicumarínicos, incluida la warfarina.

- **Toxicidad hepática:**

Las alteraciones de la función hepática, que incluyen elevación de ALT, AST y/o bilirrubina son infrecuentes con erlotinib en monoterapia.

- **Toxicidad renal:**

Las alteraciones de la función renal son infrecuentes, en caso de existir sueles ser leves o moderadas y se han relacionado con la presencia de náuseas, vómitos y diarreas que conducen a una situación de deshidratación e insuficiencia renal secundaria.

<u>En el presente estudio sólo se recogerá la información relativa a la toxicidad cutánea e intestinal, siguiendo la clasificación de la NCI (anexo 6-7, toxicidad cutánea y toxicidad intestinal).</u>

<u>Modificación de dosis</u>

Se puede llevar a cabo la reducción/ interrupción de la dosis del tratamiento debido a cualquier acontecimiento adverso en cualquier momento durante el estudio y en el caso de cualquier toxicidad que:

- No se controle mediante tratamiento médico, o
- No se tolere debido a los síntomas e interferencia con la actividad diaria normal, con independencia de la severidad.

La modificación de dosis permite dos niveles de reducción tal y como se especifica en la siguiente tabla:

Dosis inicial	Primer reducción	Segunda reducción
150 mg	100 mg	50 mg

En las dos semanas siguientes a la reducción de la dosis, la toxicidad relacionada con erlotinib debe mejorar en un grado según los criterios de NCI-CTCAE (versión 3.0) y ser menor o igual al grado 2 según NCI-CTCAE (versión 3.0), o bien se llevará a cabo otra reducción de dosis al siguiente nivel. Puede interrumpirse la administración de erlotinib durante un máximo de tres semanas si está clínicamente indicado y si la toxicidad no se controla con tratamiento médico.
Tras haber efectuado una reducción de dosis por toxicidad, no se volverá a aumentar la dosis de erlotinib, excepto en caso de toxicidad cutánea grado 3 resuelta.

Toxicidad Grado NCI-CTAE	Modificación de dosis del fármaco del estudio	Directrices para el tratamiento
	**Diarreas**	
**Grado 1**	Ninguna	Considerar loperamida
**Grado 2**	Ninguna	Loperamida
**Grado 3**	Interrupción	Interrumpir hasta resolución o grado menor o igual a 1 y reiniciar a dosis reducida
**Grado 4**	Retirada del estudio	Loperamida, fluidoterapia..
	**Exantema**	
**Grado 1**	Ninguna	Alguno de los siguientes:<br><br>Minociclina tópica<br>Tetraciclinas tópicas<br>Clindamicina tópica<br>Sufadiazina de plata tópica<br>Difenhidramina tópica<br>Esteroides tópicos<br>Esteroides orales<br>(tratamiento corto)<br><br>A criterio del investigador.
**Grado 2**	Ninguna	
**Grado 3**	Reducción de dosis, puede aumentarse cuando el exantema sea menor o igual al grado 2	
**Grado 4**	Retirada del estudio	

Pacientes del grupo 2 que reciban tratamiento con quimioterapia según BRCA1

En principio, cada paciente recibirá todos los ciclos de tratamiento a la dosis determinada al inicio del estudio, excepto en el caso de las modificaciones de la dosis previstas por razones de toxicidad. Las modificaciones de la dosis por toxicidad se efectuarán de acuerdo a las normas que se detallan a continuación.

Se permitirá un máximo de dos reducciones de dosis por paciente de cada uno de los agentes quimioterápicos.

Las modificaciones de la dosis por acontecimientos adversos efectuadas según protocolo se mantendrán reducidas en los ciclos posteriores.

Toxicidades y Modificación de la dosis:

	Cisplatino	Gemcitabina	Docetaxel
**Dosis inicial**	**75 mg/m^2 día 1**	**1250 mg/m^2 d 1& 8**	**75 mg/m^2 día 1**
**Nivel –1**	60 mg/m^2	1000 mg/m^2	65 mg/m^2
**Nivel –2**	50 mg/m^2	800 mg/m^2	50 mg/m^2
**Nivel - 3**	Suspensión	Suspensión	Suspensión

El tratamiento no podrá retrasarse más de tres semanas (≤ 21 días) por toxicidad, excepto por efectos tóxicos hematológicos, hepáticos, renales y neurológicos, en cuyo caso el tratamiento no podrá retrasarse más de dos semanas (≤ 14 días).
A continuación se describen las normas a seguir en caso de toxicidad. Todos los efectos tóxicos se clasificarán en grados utilizando los Criterios de Toxicidad Común del NCI (NCI-CTC):

- **Toxicidad hematológica**

Las dosis de docetaxel, gemcitabina y cisplatino se modificarán por neutropenia y trombocitopenia. No se practicarán modificaciones de la dosis por anemia; en esta situación, el médico aplicará el tratamiento de soporte adecuado.

1. **Modificaciones de la dosis según al Recuento Absoluto de Neutrófilos (RAN):**

Nadir del RAN x 10^9/L durante el último ciclo	Dosis a administrar<br>RAN el DÍA 1 del ciclo	
	< 1.5 x 10^9/L	≥1.5 x 10^9/L
> 1.0 ó < 1.0 durante < 7 días	Retraso[1]	Sin cambios
< 1.0 durante ≥ 7 días	Retraso[1]	Reducción
Neutropenia Febril[2] (independientemente de su duración)	Retraso[1]	Reducción

[1] *Retraso (un máximo de 2 semanas) hasta que las cifras alcancen los límites inferiores que permitan el tratamiento.*

[2] *La neutropenia febril se define como: fiebre de grado ≥ 2 (≥38.1°C), fiebre de origen desconocido sin infección documentada clínica o microbiológicamente y neutropenia de grado 3 ó 4 (<1.0 x 10^9/L), y antibióticos IV y/o hospitalización.*

2. **Modificaciones de la dosis de acuerdo a la cifra de Plaquetas:**

Nadir de la cifra de plaquetas durante el último ciclo	Dosis a administrar<br>Cifra de plaquetas el DÍA 1 del ciclo	
	< 100 x 10^9/L	≥ 100 x 10^9/L
≥ 25 x 10^9/L	Retraso[1]	Sin cambios
< 25 x 10^9/L	Retraso[1]	Reducción

[1] *Retraso (un máximo de 2 semanas) hasta que las cifras alcancen los límites inferiores que permitan el tratamiento.*

Deben determinarse semanalmente un hemograma completo con diferencial y plaquetas a fin de evaluar los efectos de la quimioterapia. El recuento absoluto de neutrófilos (RAN) deberá ser ≥ 1.5 x 10^9/L células y la cifra de plaquetas deberá ser ≥ 100 x 10^9/L antes de la administración del ciclo siguiente del tratamiento del estudio. Si debiera retrasarse la quimioterapia por toxicidad hematológica y las cifras no se recuperaran en el plazo de 2 semanas, el paciente saldrá del estudio. En el caso de que se requieran ajustes de dosis por neutropenia y trombocitopenia, sólo se realizará una reducción de dosis por ciclo de tratamiento.

- **Toxicidad hepática**

Las dosis de docetaxel y de gemcitabina se modificarán por toxicidad hepática, no así la dosis de cisplatino. Se emplearán los resultados de las pruebas de función hepática en la analítica previa a la fecha planificada de tratamiento (es decir, Día 1).

Resultados de las pruebas de función hepática					Dosis de docetaxel y gem-citabina a administrar
SGOT (AST) x LSN<br>SGPT (ALT) x LSN		Fosfatasa alcalina x LSN		Bt	
≤ 2.0	y	≤ 2.5	y	WNL	Sin cambios
> 2.0 - ≤ 5.0	y	≤ 2.5	y	WNL	Reducción[1]
> 2.0 - ≤ 5.0	y	> 2.5 - ≤ 5.0	y	WNL	Retraso[2]
> 5.0	o	> 5.0	o	>LSN	Fin tratamiento

[1]Si la toxicidad hepática empeorara (elevación ulterior de cualquiera de las pruebas hepáticas) tras la reducción de la dosis, el paciente saldrá del estudio.

[2] Retraso (un máximo de 2 semanas) hasta que las pruebas funcionales hepáticas alcancen los límites inferiores que permiten el tratamiento.

Bt, bilirrubina total

Si debiera retrasarse la quimioterapia por toxicidad hepática, se determinarán las pruebas funcionales hepáticas semanalmente hasta que éstas alcancen los límites inferiores que permiten el tratamiento. Si no se hubieran recuperado en el plazo de 2 semanas, el paciente saldrá del estudio. Una vez que se haya reducido la dosis por toxicidad hepática, si se produjera un empeoramiento posterior de las enzimas hepáticas, el paciente saldrá del estudio. Si se retrasara docetaxel o gemcitabina por toxicidad hepática, también deberá retrasarse cisplatino.

- **Toxicidad renal**

Las dosis de cisplatino se modificarán en caso de toxicidad renal. Las modificaciones de la dosis se basarán en los resultados de los exámenes en la fecha planificada de tratamiento (es decir, Día 1) de cada ciclo. No se efectuarán reducciones de la dosis de docetaxel o gemcitabina por toxicidad renal. No obstante, ambos fármacos se retrasarán si la creatinina fuera >2.0 mg/ dL.

Creatinina Sérica mg/dL		Aclaración Creatinina[1] ml/min	Dosis de docetaxel o gemcitabina	Dosis de cisplatino
**≤1.5**	**y**	**≥50**	Sin cambios	Sin cambios
**≤ 1.5**	**y**	**< 50**	Sin cambios	Retraso[2]
**> 1.5**	**y**	**< 50**	Sin cambios	Retraso[2]
**> 2.0**	**y**	**cualquiera**	Retraso	Retraso[2]

[1] La dosis de cisplatino puede reducirse utilizando el aclaramiento de creatinina calculado con la fórmula de Cockcroft-Gault o la determinación del aclaramiento de creatinina real en 24 horas.
[2] Si cisplatino se retrasara durante 1-2 semanas, la siguiente dosis se reducirá.

Si no se cumpliera el criterio renal que permite el tratamiento (aclaramiento de creatinina ≥ 50) el Día 1 del ciclo de quimioterapia, re-evaluar semanalmente. Si a las 2 semanas no se cumpliera el criterio renal, el paciente saldrá del estudio.

- **Toxicidad Neurológica**

Las dosis de docetaxel y cisplatino deberán modificarse en caso de toxicidad neurológica.

Grado de toxicidad Neurológica en la fecha planificada de tratamiento.	Dosis de docetaxel y cisplatino a administrar
**0 ó 1**	Sin cambios
**2 ó 3**	• Retraso de una semana en las dosis de cisplatino y docetaxel;<br>• Si persiste un grado ≥ 2 durante > 2 semanas, el paciente saldrá del estudio.<br>• Si el paciente se recupera a una toxicidad de grado 1, entonces reducción de ambos fármacos.<br>• Si no se recupera a un grado 1 en 2 semanas, suspensión del tratamiento.
**4**	El paciente se retira del estudio.

Si la toxicidad neurológica no fuera de grado ≤ 1 en la fecha planificada de tratamiento, éste no se administrará y el paciente se examinará a intervalos semanales. Si la toxicidad no fuera de grado ≤ 1 tras 2 semanas, el paciente saldrá del estudio

- **Reacciones de Hipersensibilidad**

A continuación se señalan la descripción y las normas de manejo de las reacciones de hipersensibilidad a docetaxel que se sugieren.
Síntomas iniciales de hipersensibilidad a docetaxel y recomendaciones de actuación

Sintomatología	Actuación médica
**Síntomas <u>leves</u>**: reacción cutánea localizada, (prurito, flushing o rash, de grado leve)	• Considerar la disminución del ritmo de infusión hasta la desaparición de los síntomas.<br>• Observación al lado del paciente.<br>• Posteriormente, completar la infusión de docetaxel al ritmo planificado inicialmente
**Síntomas <u>moderados</u>**: síntoma no listado arriba (síntomas leves) o debajo (síntomas severos), como prurito, flushing o rash generalizados, disnea, hipotensión con presión arterial (PA) sistólica > 80 mm Hg.	• Suspender la infusión de docetaxel,<br>• Administrar dexametasona IV 10 mg y/o difenhidramina 50 mg IV,<br>• Reanudar la infusión de docetaxel tras la desaparición de los síntomas
**Síntomas <u>severos</u>**, broncospasmo, urticaria generalizada, PA sistólica ≤ 80 mm Hg, angioedema	• Suspender la infusión de docetaxel;<br>• Administrar difenhidramina IV 50 mg y/o dexam IV 10 mg y/o adrenalina según se precise.<br>• Siempre que sea posible, reanudar la infusión de docetaxel en el plazo de las 3 horas siguientes a la recuperación o reinfundir al paciente en el plazo de 72 horas utilizando dexametasona 10 mg IV y/o difenhidramina 50 mg IV ½ hora antes de reanudar la infusión.<br>• Si se vuelve a producir una reacción severa a pesar la premedicación adicional, el paciente saldrá del estudio.
**<u>Anafilaxia</u>** (reacción de grado 4 del NCI)	Salida del estudio

Actitud en los siguientes ciclos de tratamiento: El pretratamiento recomendado en las infusiones posteriores consiste en 50 mg de difenhidramina por vía IV y 10 mg de dexametasona por vía IV 60 minutos antes de la infusión de docetaxel. Ello se añadirá a la dexametasona oral prescrita.

Los pacientes con reacciones de hipersensibilidad al docetaxel presentan riesgo de recurrencia de las reacciones. En los pacientes con reacciones de hipersensibilidad de carácter moderado o severo, en los ciclos posteriores el docetaxel debe administrarse a lo largo de 2 horas, junto con la premedicación arriba señalada. Estos pacientes deben ser informados acerca del riesgo potencial de presentar reacciones alérgicas recurrentes y deben ser estrechamente monitorizados.
Si la reacción inicial fuera de grado 4 de acuerdo a los Criterios de Toxicidad Común del NCI para la alergia, no se administrará más tratamiento al paciente y éste saldrá del estudio. Si se produjera una segunda reacción severa (grado 3) a pesar de las premedicaciones adicionales antes descritas, el paciente saldrá del estudio.
En el caso de síntomas tardíos de hipersensibilidad, como aparición en el plazo de 1 semana del tratamiento de prurito localizado o generalizado, podrá administrarse tratamiento sintomático (por ej., un antihistamínico oral). Dependiendo de la intensidad de la reacción observada, en el siguiente ciclo de tratamiento podrá administrarse premedicación oral o IV con un antihistamínico. En ningún caso se efectuarán reducciones de la dosis.

- **Toxicidad auditiva**

Cisplatino puede provocar una pérdida auditiva de las frecuencias altas. Si se produjera una pérdida auditiva de grado 1/2, deberá evaluarse el riesgo de una pérdida auditiva adicional frente al beneficio potencial de continuar el tratamiento con cisplatino. Una pérdida auditiva de grado 3/4 supone una indicación para la suspensión del fármaco.

- **Náuseas y vómitos**

Las náuseas y/o los vómitos deben controlarse mediante los antieméticos adecuados. Si se produjeran náuseas/vómitos de grado 4 a pesar de los antieméticos, en el siguiente ciclo deberá reducirse la dosis de cisplatino.

- **Mucositis**

En caso de mucositis en la fecha planificada de tratamiento (Día 1), éste se retrasará hasta que se produzca la recuperación. Si, en cualquier momento, se produce una mucositis aguda de grado 3/4, la siguiente dosis de docetaxel, gemcitabina y cisplatino deberá reducirse un nivel y reanudarse el tratamiento tras la recuperación.

- **Diarrea**

Deberán administrarse los tratamientos sintomáticos adecuados. Si diarrea grado 3/4, la siguiente dosis de docetaxel, gemcitabina y cisplatino se retrasará hasta recuperación, y se reanudará con disminución de un nivel de dosis.

- **Retención de líquidos**

No se han planificado reducciones de la dosis de docetaxel en caso de retención de líquidos.

Los pacientes que presenten por vez primera un edema o éste sea sintomático, o con otros signos de aumento de la retención de líquidos, deberán recibir tratamiento con diuréticos orales. A continuación se señalan aquellos regímenes que han mostrado eficacia en el tratamiento de la retención de líquidos debida a docetaxel. A criterio del investigador, el tratamiento diurético podrá iniciarse en el orden que se señala:

- Espironolactona, 50 mg al día, que se puede aumentar hasta tres veces al día..
- Furosemida, 40 mg PO al día, si no responde a espironolactona. Pueden administrarse suplementos de potasio según se precise.
- Si, tras un período de prueba ≥ 2 semanas, este tratamiento no fuera eficaz, administrar furosemida 20 mg PO al día más metolazona 2,5 mg PO al día con suplementos de potasio según se precise.

El tratamiento ulterior dependerá de la situación clínica. El criterio médico del investigador determinará si lo mejor para el paciente será continuar o abandonar el tratamiento. En caso de retención de líquidos severa que no responda al tratamiento sintomático, el paciente saldrá del estudio.

- **Otras toxicidades no especificadas anteriormente**

En caso de toxicidades de grado ≤ 2, tratamiento sintomático, si es posible, y volver a tratar sin reducción de la dosis.
En caso de toxicidades de grado ≥ 3, se suspenderá el fármaco sospechoso hasta la resolución del efecto tóxico a grado ≤ 1 o a la situación basal, si aquella hubiera sido mayor de grado 1; a continuación, reinstitución, si fuera médicamente adecuado, con reducción de un nivel de dosis.

- **Modificaciones de la dosis de Gemcitabina el día 8:**

1. Modificaciones de la dosis de gemcitabina el día 8 por toxicidad hematológica:

RAN x 10^9/L		Recuento de plaquetas x 10^9/L	Dosis de gem
**≥ 1.5**	y	**≥ 100**	Sin cambios
**1.0 – 1.499**	y/o	**75 – 99**	Reducción al Nivel -2
**< 1.0**	y/o	**< 75**	Retraso

Modificaciones de la dosis de Gemcitabina el día 8 por Toxicidad No-Hematológica:

Grado NCI-CTC	Dosis de Gemcitabina
**0 – 2 (y náuseas o vómitos grado 3)**	Sin cambios
**3 (excepto náuseas/vómitos)**	Reducción al Nivel –2
**4**	No administrar esa dosis

ANEXO 1: HOJA DE INCLUSIÓN Y ASIGNACIÓN TRATAMIENTO

ENVIAR POR FAX: a la atención de Esther Carrasco Chaumel/ Mireia Tomás Solá
FAX: 93 497 89 50 TELÉFONO: 93 497 89 25 MÓVIL: 616 999 881

Spanish Lung Adenocarcinoma Trial **SLAT**

HOSPITAL:____

INVESTIGADOR:____

TELÉFONO DE CONTACTO:_______________ FAX:__________________

e-mail contacto del investigador: __________________________________

NÚMERO DE HISTORIA CLÍNICA: ____________________

INICIALES DEL PACIENTE: ____ ____ ____

FECHA DE NACIMIENTO: ___ ___ /___ ___ / ___ ___ ___ ___

CRITERIOS DE INCLUSIÓN: SI □ NO □

CRITERIOS DE EXCLUSIÓN: SI □ NO □

¿HA FIRMADO EL PACIENTE LA HOJA DE CONSENTIMIENTO INFORMADO?
SI □ NO □

¿SE HA ENVIADO:
- PIEZA DEL TUMOR SI □ NO □
- HEMATOXILINA /EOSINA SI □ NO □
- INFORME DE AP SI □ NO □

FECHA DE ENVÍO DE FAX: __ __ /__ __ / __ __

**NO CUMPLIMENTAR**			
**MUESTRA SUFICIENTE:**	SI □ NO □		
**MUTACIONES DE EGFR**		**EXPRESIÓN DE BRCA1**	
NO □ SI □ ⇨ **Enviar muestras de sangre**		Bajo □ Medio: □ Alto □	
**TRATAMIENTO ASIGNADO**			
Erlotinib □	Gem/cis □	Doc/cis □	Doc □

CÓDIGO DE PACIENTE ASIGNADO: **AT ___ ___ ___ ___**
FECHA: __ __ /__ __ /__ __ __ __ FIRMA:

ANEXO 2: CONSENTIMIENTO INFORMADO

DOCUMENTO DE AUTORIZACIÓN PARA EL ANÁLISIS DE TEJIDO TUMORAL Y SANGRE

Tratamiento individualizado en función de las mutaciones en EGFR y del nivel de expresión de BRCA1 en pacientes con adenocarcinoma de pulmón avanzado *Spanish Lung Adenocarcinoma Trial (SLAT)*

Estimado/a paciente:

Queremos solicitar su autorización para utilizar muestras de su tejido pulmonar tumoral y de sangre para realizar una serie de pruebas moleculares en ellos. El objeto de este estudio es saber si la existencia de determinadas alteraciones influyen en la respuesta al tratamiento y en la evolución de su enfermedad.

Si decide dar su autorización para el uso de su tejido tumoral y sanguíneo, le pediremos a su médico que nos entregue una parte de ese tejido que está almacenado en el hospital dónde se realizó el diagnóstico. En cuanto a la obtención de sangre, se le extraerán 10 ml más de sangre (un tubo más) dentro de las extracciones previstas por su médico. Ninguna de estas pruebas aumentará su número de visitas al hospital ni suponen ningún riesgo, molestia ni incomodidad adicional para usted.

El resultado de las determinaciones antes mencionadas se comparará con su información médica con el objeto de conocer más detalles sobre la biología molecular de su tumor y/o sobre la eficacia que el tratamiento puede tener sobre usted.

El manejo de la muestra se realizará de forma totalmente anónima, sin que los científicos que se encargan del análisis, sean capaces de identificarle a Usted. Todos sus datos (personales, clínicos y datos que procedan de la muestra de tumor y de su sangre) serán tratados de acuerdo con la “directiva de la protección de los sujetos con respecto al procesamiento de los datos personales” y la Ley Orgánica 15/1999, de 13 de diciembre, de protección de datos de carácter personal.”

La decisión de dar autorización para la determinación de estas pruebas en sus muestras es totalmente voluntaria. Puede negarse a dar dicha autorización o a denegarla en cualquier momento sin que esto suponga ninguna penalización ni beneficios a los que tiene usted derecho en la asistencia clínica que se le presta habitualmente.

Es consciente de que esta autorización incluye también su consentimiento para permitir que el resultado pueda ser registrado, utilizado para su análisis científico por el personal médico que le atiende, personas autorizadas por él o por las autoridades nacionales y/o internacionales, y presentado en congresos, reuniones científicas y publicaciones médicas, siempre respetando su anonimato.

Yo, ____, con fecha de nacimiento ____________________

AUTORIZO, libre y voluntariamente, a que se estudie la presencia de una serie de biomarcadores en mis muestras de tejido tumoral y en las muestras sanguíneas.

Firma del paciente: .... Fecha:…………………………………

Firma del investigador:........................... Fecha: …………………………………..

Nombre del investigador : ………………………………………………………………………………………..

Centro hospitalario: ……………………………………………………………………………………………..

ANEXO 3: DATOS PARA EL ENVÍO DE MUESTRAS Y CRDs

SLAT (Spanish Lung Adenocarcinoma Trial)

Data Managers: Esther Carrasco Chaumel/ Mireia Tomás Solá
Servicio de Oncología Médica-ICO
Hospital Universitari Germans Trias i Pujol
Carretera del Canyet s/n
08916 Badalona (Barcelona)
FAX: 93.4978950
Teléfono: 93. 4978925
Móvil data managers: 616.999.881/ 650.172.172
E -mail: data managers <slatdata@can-ruti.e.telefonica.net>

SERVICIO DE MENSAJERÍA:
MRW

Número de abonado: # 15062 /Mutaciones # 15062 #
Teléfono 93.460.02.22

ANEXO 4: CENTRIFUGACIÓN DE LAS MUESTRAS DE SANGRE

SOLO DEBE REALIZARSE EN AQUELLOS PACIENTES CON MUTACIONES POSITIVAS PARA EGFR

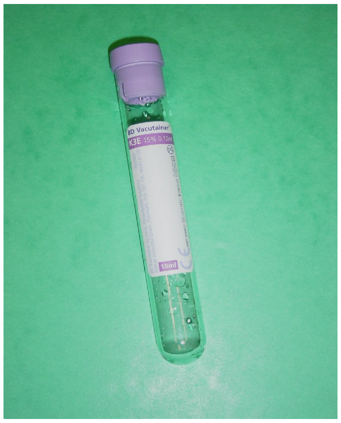

1 tubo x EDTA/K3 → Enviar sin centrifugar (10 ml sangre)

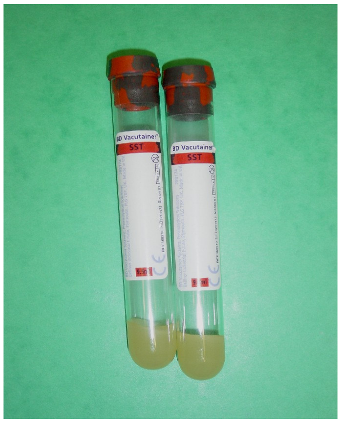

2x SST GEL (20 mL)

Dejar 15-20 min. en reposo y posición vertical a temperatura ambiente

↓

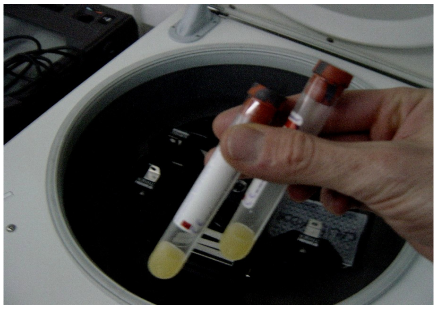

Centrifugar 1.800 rpm x 10'

↓

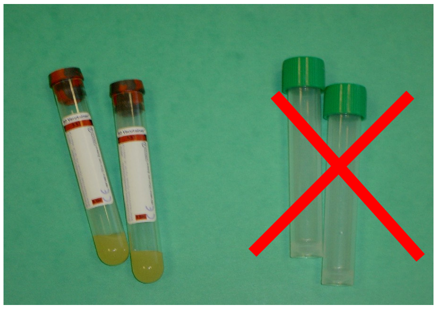

Transferir S/N (suero) a tubo limpio estéril y desechar el coagulo

ANEXO 5: ENVIO DE MUESTRAS

LA SANGRE SOLO DEBE ENVIARSE EN AQUELLOS PACIENTES QUE PRESENTEN MUTACIONES PARA EGFR

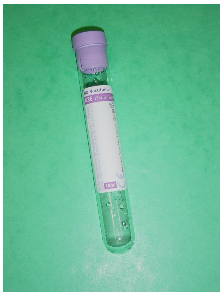

+

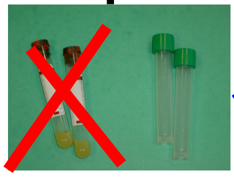

Enviar a Tª Ambiente

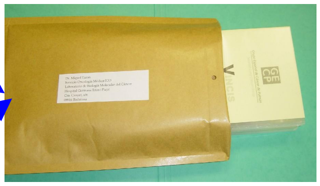

Courier Service
MRW: 93.460.02.22
# 15062/mutaciones #

Servicio Oncología Médica-ICO
Hospital Germans Trias i Pujol
Ctra Canyet s/n
08916-Badalona (Spain)

Dr. Rafael Rosell
Dr. Miquel Taron 629.672.819
Dra. Noemí Reguart 690.852.730
D.M Esther Carrasco 616.999.881
D.M Mireia Tomás 650.172.172

Bloque de tejido parafinado

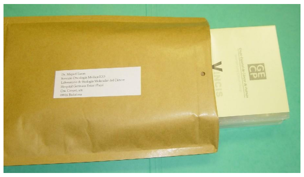

ANEXO 6: HOJA DE TOCICIDADES GASTROINTESTINALES NCI

	0	1	2	3	4
**Colitis**	none	(-)	Abdominal pain with mucus and/ or blood in stool.	Abdominal pain, fever, change in bowel habits with ileus or peritoneal signs, and radiographic or biopsy documentation	Perforation or requiring surgery or toxic megacolon
**Diarrhea**	none	Increase of < 4 stools/ day over pre-treatment	Increase 4-6 stools/ day, or nocturnal stools	Increase > 7 stools/ day or incontinence, or need for parenteral support for dehydratation	Physiologic consequences requiring intensive care; or hemodynamic collapse

ANEXO 7 HOJA DE TOCICIDADES CUTANEAS NCI

	0	1	2	3	4
**Bruising (in absence of grade 3 or 4 thrombocytopenia)**	none	Localized or in dependent area	generalized	(-)	(-)
**Dry skin**	normal	Controlled with emollients	Not controlled with emollients	(-)	(-)
**Erythema multiforme**	absent		Scattered but not generalized eruption	Severe or requiring IV fluids (e.g. generalized rash or painful stomatitis)	Life-threatening (exfoliative or ulcerating dermatitis or requiring enteral or parenteral nutritional support)
**Flushing**	Absent	Present	(-)	(-)	(-)
**Hand-foot skin reaction**	none	Skin changes or dermatitis without pain (eg. Erythema, peeling)	Skin changes with pain, not interfering with function	Skin changes with pain, interfering with function	(-)
**Photosensitity**	none	Painless erythema	Painful erythema	Erythema with desquamation	(-)
**Pruritus**	none	Mild or localized, relieved spontaneously or by local measures	intense or widespread, relieved spontaneously by systemic measures	Intense or widespread and poorly controlled despite treatment	(-)
**Rash/ desquamation**	none	Macular or papular eruption or erythema without associated symptoms	Macular or popular eruption or erythema without pruritus or other associated symptoms covering >25% - <50% of body surface or localized desquamation or other lesions covering >25- <50% of body surface area	Symptomatic generalized erythrodermia or symptomatic macular, popular or vesicular eruption, without bullous formation or desquamation covering > 50% of body surface area.	Generalized exfoliative dermatitis or ulcerative dermatitis or bullous formation
**Urticaria**	none	Requiring no medication	Requiring PO or topical treatment or IV medication or steroids for <24 hours	Requiring IV medication or steroid for > 24 hours.	
**Dermatitis / Skin, other toxicities**	none	mild	moderate	severe	Life-threating

ANEXO 8 PETICIÓN DE MEDICACIÓN COMPASIVA

La dirección dónde se debe la bibliografía para uso compasivo es

madrid.biomednet@roche.com

Estudio SLAT/SLADB

Adenocarcinoma en primera linea de tratamiento con enfermedad avanzada y mutaciones positivas para EGFR
