# Supplementary material for: Customized Treatment in Non-Small-Cell Lung Cancer Based on EGFR Mutations and BRCA1 mRNA Expression
Source: PLoS One. 2009 May 5;4(5):e5133. doi: 10.1371/journal.pone.0005133 (PMC2673583; doi:10.1371/journal.pone.0005133)
Supplement: Protocol S2 — English summary of protocol (0.21 MB PDF) [file pone.0005133.s013.pdf]

Those researchers interested can participate in the SLAT study:

Spanish Lung Adenocarcinoma Trial (SLAT)

Study of personalised treatment in accordance with EGFR mutations and the level of BRCA1 in patients with advanced lung adenocarcinoma

In which a second group of patients not carrying EGFR mutations is set up.

Patients not carrying Epidermal Growth Factor Receptor (EGFR) mutations (Group 2) shall receive **individual chemotherapy treatment in accordance with the levels of BRCA 1 m RNA. Depending on these levels, three different treatment subgroups shall be established:**

- Subgroup A with low levels of BRCA1 mRNA shall receive **gemcitabine/cisplatin treatment**
- Subgroup B with medium levels of BRCA1 mRNA: shall receive **docetaxel/cisplatin treatment**
- Subgroup C with high levels of BRCA1 mRNA: shall receive **docetaxel treatment**

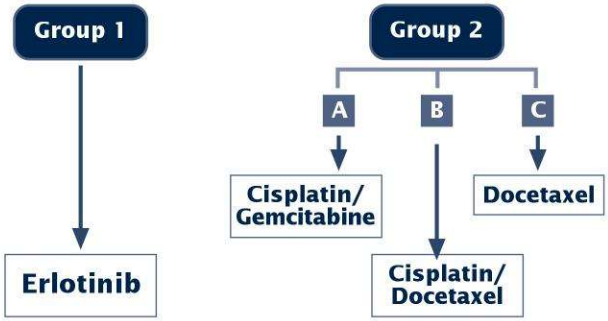

Algorithm of treatment for adenocarcinomas in accordance with the established levels of EGFR and BRCA1, distribution percentages and estimated survival times per treatment group.

Adenocarcinoma

EGFR TK mutations & BRCA1 mRNA

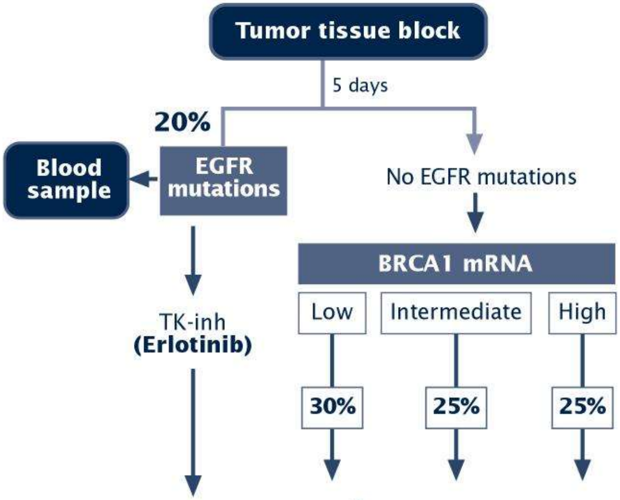

	Erlotinib	gem / cis	doc + cis	doc
MS	36 mo	18 mo	15 mo	>12 mo
3-yr	50%	30%	25%	20%
